# The orphan receptor TR3 participates in angiotensin II-induced cardiac hypertrophy by controlling mTOR signalling

**DOI:** 10.1002/emmm.201201369

**Published:** 2012-11-29

**Authors:** Rong-Hao Wang, Jian-Ping He, Mao-Long Su, Jie Luo, Ming Xu, Xiao-Dan Du, Hang-Zi Chen, Wei-Jia Wang, Yuan Wang, Nan Zhang, Bi-Xing Zhao, Wen-Xiu Zhao, Zhong-Gui Shan, Jiahuai Han, Chawnshang Chang, Qiao Wu

**Affiliations:** 1 State Key Laboratory of Cellular Stress Biology, School of Life Sciences, Xiamen UniversityXiamen, Fujian Province, China; 2 Xiamen Heart Center, Zhongshan Hospital, Xiamen UniversityXiamen, Fujian Province, China; 3 Department of Physiology, Fourth Military Medical UniversityXian, China; 4 George H. Whipple Lab for Cancer Research, Departments of Pathology and Urology and the Cancer Center, University of Rochester Medical CenterRochester, NY, USA

**Keywords:** angiotensin II, cardiac hypertrophy, mammalian target of rapamycin, orphan receptor TR3, tuberous sclerosis complex

## Abstract

Angiotensin II (AngII) induces cardiac hypertrophy and increases the expression of TR3. To determine whether TR3 is involved in the regulation of the pathological cardiac hypertrophy induced by AngII, we established mouse and rat hypertrophy models using chronic AngII administration. Our results reveal that a deficiency of TR3 in mice or the knockdown of TR3 in the left ventricle of rats attenuated AngII-induced cardiac hypertrophy compared with the respective controls. A mechanistic analysis demonstrates that the TR3-mediated activation of mTORC1 is associated with AngII-induced cardiac hypertrophy. TR3 was shown to form a trimer with the TSC1/TSC2 complex that specifically promoted TSC2 degradation via a proteasome/ubiquitination pathway. As a result, mTORC1, but not mTORC2, was activated; this was accompanied by increased protein synthesis, enhanced production of reactive oxygen species and enlarged cell size, thereby resulting in cardiac hypertrophy. This study demonstrates that TR3 positively regulates cardiac hypertrophy by influencing the effect of AngII on the mTOR pathway. The elimination or reduction of TR3 may reduce cardiac hypertrophy; therefore, TR3 is a potential target for clinical therapy.

## INTRODUCTION

It is well known that hypertension can cause cardiac hypertrophy. Persistent hypertrophy leads to heart failure, which is the primary cause of mortality worldwide (Rame & Dries, 2007). Angiotensin II (AngII) is produced in peripheral tissues and increases blood pressure (BP) by stimulating the kidney to release aldosterone, which then induces water and salt reabsorption (Sachse & Wolf, 2007). In addition, AngII contributes to the development of cardiovascular diseases by causing hypertension, cardiac hypertrophy, myocardial fibrosis, thrombosis, endothelial dysfunction and even organ damage. In cardiomyocytes, AngII triggers molecular pathways that promote protein synthesis, enhance the production of reactive oxygen species (ROS) and induce apoptosis by binding to its receptors: angiotensin II type I receptor (AT1R) and angiotensin II type II receptor (AT2R). The physiological effect of AngII is mainly transduced by its binding with AT1R. AT1R transgenic mice have been shown to exhibit enlarged hearts compared with controls (Paradis et al, 2000); however, AT2R transgenic mice demonstrate no obvious changes in heart size (Sugino et al, 2001). Thus, many anti-hypertrophic drugs have been designed to antagonize AT1R. For example, the well-known AT1R antagonists losartan, valsartan and telmisartan are used in the clinical therapy of cardiac hypertrophy.

The mammalian target of rapamycin, mTOR, is widely understood to have an important role in cardiac hypertrophy because of its capacity to regulate cell growth (Gao et al, 2006). mTOR has two distinct complexes, which are referred to as mTORC1 and mTORC2. mTORC1 consists of mTOR, Raptor, Deptor, mLST8 and PRAS40 and regulates various cellular characteristics, such as cell size, cell cycle, cell survival and autophagy, in a manner that is dependent on its downstream substrates, such as S6K1 and 4E-BP1 (Burnett et al, 1998). The activation of mTORC1 requires its recruitment to the endomembrane, which is mediated by Rag GTPases. In the endomembrane, Rheb, which is a Ras-related GTP protein, activates mTOR, although the detailed mechanism of its action remains unclear. The activity of Rheb is regulated by the TSC1/TSC2 complex. TSC1 stabilizes TSC2, and TSC2 acts as a GTPase-activating protein (GAP) to inactivate Rheb by removing its bound GTP, thereby negatively regulating mTOR activity (Sancak et al, 2010). It is widely known that some environmental stimuli require the TSC complex to regulate mTORC1 signalling. During this process, AMPK, AKT, and ERK affect mTORC1 activity by regulating the stability of TSC2. Unlike mTORC1, mTORC2 is rapamycin-resistant. mTORC2 contains mTOR, Rictor, mLST8, PRR5 and mSin1. As a kinase, mTORC2 mainly phosphorylates the AGC family (including AKT, PKCα and SGK1) to affect glucose regulation and cytoskeletal organization (Lamming et al, 2012).

TR3 (also termed Nur77) is a member of the steroid/thyroid/retinoid receptor family (Germain et al, 2006) and has dual functions in cell growth and death. Although its role in cell death that occurs mainly through the apoptotic pathway is prevalent in many types of cancer cells (Chen et al, 2012b; Yao et al, 2012; Zhao et al, 2011), TR3 is also thought to promote the proliferation of many normal cell types, including adipocytes (Fumoto et al, 2007) and vascular endothelial cells (Zeng et al, 2006). As a transcription factor, TR3 induces the expression of many growth-related genes. For example, TR3 promotes proliferation by regulating the gene expression of cyclin D and cyclin E in preadipocytes (Fumoto et al, 2007). Recently, we demonstrated that the isomerization by Pin1 enhances TR3 transcriptional activity to promote cell proliferation (Chen et al, 2012a). However, the participation of TR3 in the regulation of cell size remains largely unclear.

AngII has been reported to induce TR3 expression in human, bovine and rat adrenocortical cells (Kelly et al, 2004). AngII also plays a role in the development of cardiac hypertrophy, which suggests that TR3 may participate in pathological AngII-induced cardiac hypertrophy. To explore the role of TR3 in cardiac hypertrophy and to determine which signalling pathway is involved, we established AngII-induced cardiac hypertrophic mouse and rat models by increasing the BP of animals. In these two models, we excluded the possibility that TR3 expression was correlated with the increase of BP and observed that the heart enlargement induced by AngII was significantly attenuated in TR3 knockout (TR3-KO) mice and in rats with knockdown of TR3 in the left ventricle (TR3-KD) compared to the respective wild-type (WT) mice or rats. TR3-KO mice and TR3-KD rats exhibited relatively lower risk of cardiac dysfunction, as determined by fibrosis, apoptosis and heart failure. Further mechanistic analysis indicated that this phenomenon might result from the control of mTORC1 signalling by TR3. TR3 bound to TSC2-TSC1 dimers and induced TSC2 ubiquitination and degradation, which led to the activation of mTORC1 but not mTORC2. When mTORC1 was activated, the size of cardiomyocytes was enlarged, protein synthesis was enhanced and the level of ROS was increased. In clinical studies, patients with left ventricular thickness exhibited higher levels of TR3 expression and an associated increased degradation of TSC2. Therefore, this study provides insight into the mechanism by which TR3 regulates AngII-induced pathological hypertrophy through its influence on mTOR signalling and reveals that TR3 may be a unique target for the treatment of clinical cardiac hypertrophy.

## RESULTS

### TR3 mediates AngII-induced cardiac hypertrophy in mice

**Figure 1:**
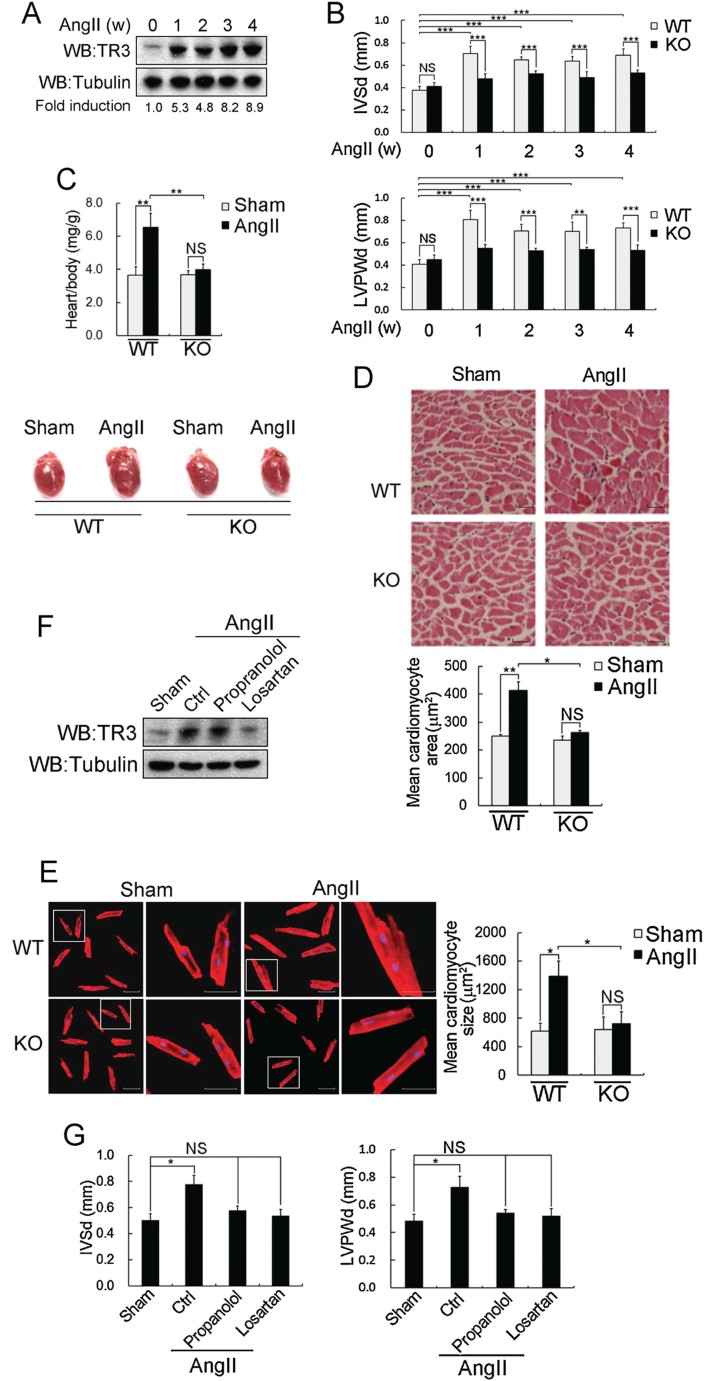
TR3-mediated AngII-induced cardiac hypertrophy in mice In the AngII-induced hypertrophy model, AngII was continuously provided for 4 weeks using an osmotic minipump. The same volume of 0.9% saline including 0.01 M acetic acid was substituted for AngII in sham-operated mice. AngII elevated TR3 protein levels. The left ventricles from individual hearts (*n* = 5 per group) were removed, and the proteins were extracted. The expression levels of TR3 were determined by Western blotting using a TR3 antibody followed by quantification (bottom: fold induction). Tubulin was used as a loading control.Echocardiographic analysis of WT mice and TR3-KO littermates (*n* = 15 per group). IVSd and LVPWd were measured weekly.The heart weight to body weight (mg/g) ratios (top panel) (*n* = 5 per group) and representative heart images (bottom panel) from 4-week sham-operated and AngII-treated mice. Hearts were dissected from the euthanized mice, weighed and normalized to body weight.Cardiomyocyte size in left ventricle sections of the sham-operated or AngII-treated WT and KO mice. H&E staining was performed (top), and the mean cross-sectional area was calculated using ImageJ (bottom) (scale bar = 50 µm).The size of cardiomyocytes isolated from the hearts of WT and TR3-KO adult mice. The mice were treated with AngII for 4 weeks using implanted osmotic minipumps. The cardiomyocytes were isolated as described in the Supporting Information Methods. The cardiomyocytes were stained with an anti-α-actinin antibody (left), and the mean cardiomyocyte size was calculated using ImageJ (right).AngII upregulates TR3 expression through its own receptor. During the administration of AngII, the mice (*n* = 5 per group) received losartan (200 mg/L) or propranolol (100 mg/L) in their drinking water for 2 weeks. The left ventricles were removed, and the proteins were extracted for the analysis of TR3 expression by Western blotting.IVSd and LVPWd were measured in the same mice as in Fig 1F. In each panel shown above, the following symbols were used: ***p < 0.001, **p < 0.01, *p < 0.05 and NS: non-significant.

A recent study demonstrated increased TR3 expression in the transverse aorta constriction (TAC) mouse model, which can induce pathological cardiac hypertrophy (Cheng et al, 2011). Similar to TAC, AngII has been reported to cause pathological cardiac hypertrophy. Therefore, we investigated whether TR3 is involved in AngII-induced cardiac hypertrophy. To establish a model of AngII-induced cardiac hypertrophy, 12-week-old WT mice and age-matched TR3-knockout (TR3-KO) littermates were implanted with osmotic minipumps to administer AngII continuously for 4 weeks. TR3 expression in the WT mice and the BP of mice with both genotypes were upregulated within 4 weeks. However, the increase in BP by AngII was comparable between the WT and TR3-KO mice (Fig 1A and Supporting Information Fig S1A). These results suggest that TR3 expression, rather than BP, may cause the difference in phenotype between the WT and TR3-KO mice.

Indices for the development of cardiac hypertrophy, including the interventricular septum diastolic (IVSd) and left ventricular posterior wall thickness diastolic (LVPWd) dimensions, were measured weekly by echocardiography. The IVSd and LVPWd values significantly increased in the WT mice following the administration of AngII but increased only slightly in the TR3-KO mice (Fig 1B), indicating that TR3 enhances AngII-induced cardiac hypertrophy. The remaining echocardiographic parameters are summarized in Table 1. Heart rate (HR) and BP remained comparable between the WT and TR3-KO mice before and after AngII administration. However, in agreement with the changes of the IVSd and LVPWd, the diastolic and systolic left ventricular internal dimensions (LVIDd and LVIDs) decreased in the WT mice but not in the TR3-KO mice after the administration of AngII. The fractional shortening (FS), which reflects the contractile function of the heart, was significantly reduced from 65.89 to 31.46% in the WT mice but was maintained in the KO mice (67.89–62.42%) after AngII administration. In the TR3-KO mice, heart size (Fig 1C, bottom) and the heart weight to body weight ratio (Fig 1C, top) were not obviously changed before and after AngII administration. However, both of these parameters were markedly increased in the WT mice, which can be attributed to increased cell volume. Histopathological analysis with H&E staining clearly indicated that after AngII administration, the mean cross-sectional area of the cardiomyocytes from the left ventricle was greater in WT mice than in TR3-KO mice, whereas no differences were observed between the sham-operated WT and TR3-KO mice (Fig 1D). Because the heart contains cardiomyocytes and non-cardiomyocytic cells, we used histochemistry to analyse the AngII-induced TR3 expression in the cardiomyocytes of the heart tissue of mice. TR3 was not only colocalized with tropomyosin-positive cardiomyocytes but was also elevated by AngII in the cytoplasm (Supporting Information Fig S1B, top). Immunofluorescent analysis further confirmed this finding (Supporting Information Fig S1B, bottom). Furthermore, we also observed enlarged cells among fresh cardiomyocytes isolated from adult WT mice when the mice were treated with AngII for 4 weeks, whereas the cardiomyocytes isolated from adult TR3-KO mice showed no obvious changes after AngII treatment (Fig 1E). Taken together, these data strongly suggest that TR3 is required for AngII-induced cardiac hypertrophy.

**Table 1:** Ecocardiographic paramters from sham-operated and AngII-administrated mice

	TR3-WT		TR3-KO	
		
	Sham (*n* = 15)	AngII (*n* = 15)	Sham (*n* = 15)	AngII (*n* = 15)
IVSd (mm)	0.37 ± 0.03	0.68 ± 0.05 (p1 ***, p2 ***)	0.41 ± 0.03	0.53 ± 0.02
LVPWd (mm)	0.40 ± 0.04	0.74 ± 0.04 (p1 ***, p2 ***)	0.45 ± 0.39	0.53 ± 0.04
LVIDd (mm)	2.29 ± 0.22	1.65 ± 0.29 (p1 *, p2 *)	2.26 ± 0.46	2.11 ± 0.22
LVIDs (mm)	1.70 ± 0.35	1.14 ± 0.11 (p1 **, p2 *)	1.67 ± 0.22	1.68 ± 0.20
FS (%)	65.89 ± 5.72	31.46 ± 4.01 (p1 ***, p2 ***)	67.89 ± 5.07	62.42 ± 4.80
HR (bpm)	635.21 ± 53.73	628.01 ± 35.65	642.97 ± 13.24	622.44 ± 29.70
BP (mm Hg)	87.50 ± 8.88	121.75 ± 7.41	85.21 ± 9.54	121.50 ± 8.88
BW (g)	30.52 ± 0.32	27.95 ± 0.68	30.91 ± 2.38	29.16 ± 0.84
HW (g)	0.1105 ± 0.0138	0.1820 ± 0.0210 (p1 **, p2 **)	0.1137 ± 0.0142	0.1162 ± 0.0117
TL (mm)	17.5 ± 0.6	17.0 ± 0.8	17.7 ± 0.9	18.0 ± 0.8
BW/TL (g/mm)	1.74 ± 0.11	1.64 ± 0.07	1.74 ± 0.10	1.62 ± 0.04
HW/TL (mg/mm)	6.31 ± 0.75	10.70 ± 1.02 (p1 **, p2 **)	6.41 ± 0.80	6.46 ± 0.62

IVSd, indicates diastolic interventricular septum; LVPWd, diastolic left ventricular posterior wall thickness; LVIDd, diastolic left ventricular internal dimension; LVIDs, systolic left ventricular internal dimension; FS, fractional shortening; HR, heart rate; BP, blood pressure; BW, body weight; HW, heart weight; TL, tibial length.

After AngII administration for 4 weeks, TR3-WT mice exhibited increased length of IVSd and LVPWd and reduced LVIDd and LVIDs. FS in TR3-WT mice decreased to 31.46%, but in TR3-KO mice, FS was not affected (62.42%) by AngII administration. Sham-operated and AngII-operated TR3-WT and TR3-KO mice were weighed, and their hearts were removed and measured. For HW measurement, all hearts were dried at 60°C and weighed. Tibias were dissected and measured. Comparisons were made between TR3-WT and TR3-KO after AngII administration using the ANOVA statistical method. p1, WT *versus* TR3-KO after AngII administration; p2, sham-operation *versus* AngII administration in WT mice. ***p < 0.001; **p < 0.01; *p < 0.05.

To determine whether the up-regulation of TR3 expression is directly caused by AngII and whether this upregulation is uncorrelated with hypertension, losartan (an AngII receptor antagonist) and propranolol (a blocker of hypertension) were used to treat WT mice during the administration of AngII. Although losartan and propranolol both ameliorated the AngII-induced high BP (Supporting Information Fig S1C), only losartan efficiently blocked the AngII-induced expression of TR3 (Fig 1F). Moreover, regardless of the expression of TR3, the mice that were subjected to losartan or propranolol treatment did not exhibit increased IVSd and LVPWd, even when administered AngII (Fig 1G). Therefore, it is likely that the upregulation of TR3 occurs directly through AngII signalling in cardiomyocytes but not through AngII-induced hypertension.

### TR3 knockout mice and rats with organ-specific knockdown of TR3 display reduced AngII-induced cardiac dysfunction

**Figure 2:**
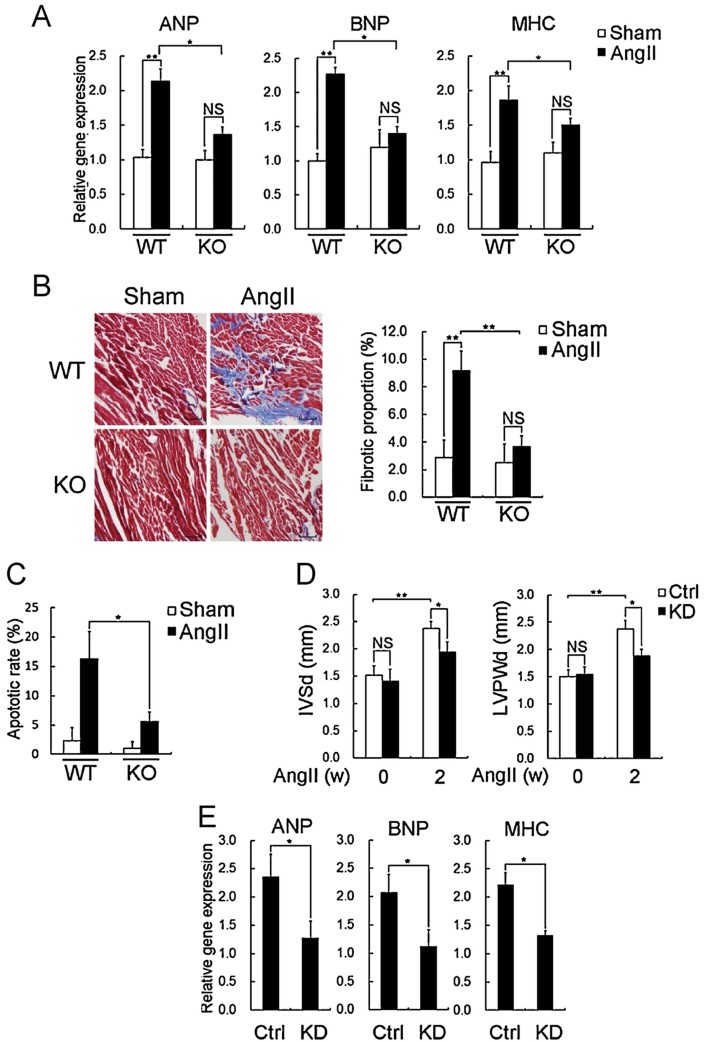
TR3 knockout mice (TR3-KO) or rats with knockdown of TR3 in the left ventricle (TR3-KD) had less cardiac dysfunction following AngII treatment compared with the respective controls The mice were sham-operated or treated with AngII for 4 weeks as described above. The rats were treated with AngII for 2 weeks. Gene expression analysis of *ANP*, *BNP* and *MHC* in the WT and TR3-KO mice. Total RNA was isolated from the left ventricles of the mice (*n* = 5 per group). Real-time PCR analysis was performed to determine the expression of the indicated genes.Masson's trichrome staining shows cardiac fibrosis. A Masson's trichrome staining kit was used to visualize deposited collagen in the heart sections. The area of cardiac fibrosis was quantified using ImageJ (scale bar = 50 µm).TUNEL staining was used to detect apoptotic cells in the heart sections. Apoptotic cells were counted in three independent sections, and 300 nuclei were calculated in each section.IVSd and LVPWd were measured in the WT and TR3-KD rats before and after the administration of AngII (*n* = 5 per group).Gene expression levels of *ANP*, *BNP* and *MHC* in the WT and TR3-KD rats. Total RNA was isolated from the left ventricles of the rats (*n* = 5 per group). Real-time PCR analysis was then performed to determine the expression levels of the indicated genes. **p < 0.01, *p < 0.05 and NS: non-significant.

AngII-induced cardiac hypertrophy is accompanied by a series of pathological changes, including apoptosis, fibrosis and the reprogramming of certain fetal genes (Braunwald & Bristow, 2000; Rame & Dries, 2007). Therefore, we measured the mRNA levels of *ANP*, *BNP* and *MHC*, which are often used as markers of pathological cardiac hypertrophy. Treatment with AngII effectively increased the mRNA levels of these three genes in the WT mice but not in the TR3-KO mice (Fig 2A). To further characterize the cardiac dysfunction caused by hypertrophy, we measured fibrosis and apoptosis, both of which are responsible for heart failure (Diez et al, 1997; Swynghedauw, 1998). The administration of AngII caused marked cardiac fibrosis in the hearts of the WT mice compared with those of the TR3-KO mice (Fig 2B). Additionally, more apoptotic cells were found in the hearts of the WT mice than in those of the TR3-KO mice following the AngII treatment; the rate of apoptosis increased from approximately 2–16% following the AngII treatment in the WT mice (Fig 2C).

To demonstrate the organ-specific role of TR3 in cardiac hypertrophy *in vivo*, we established another animal model in which endogenous TR3 was specifically knocked down in the left ventricle of the rats but not in other tissues (including the right ventricle, liver, muscle and kidney) by injecting lentivirus-packed siTR3 into the left ventricle (Supporting Information Fig S2A). After the knockdown of TR3, the rats, termed TR3-KD, were administered AngII using osmotic minipumps for 2 weeks. During this period, the TR3-KD rats and control rats (injected with scrambled-siRNA) exhibited similar levels of hypertension (Supporting Information Fig S2B). The IVSd and LVPWd values obtained from the echocardiographic measurements were lower in the TR3-KD rats than in the control rats when AngII was used to treat the rats for 2 weeks (Fig 2D). Moreover, the levels of the *ANP*, *BNP* and *MHC* transcripts were significantly different between the TR3-KD rats and the control rats after the AngII infusion (Fig 2E). These data indicate a specific role for TR3 in the heart and demonstrate that decreased TR3 expression in the heart may provide protection against AngII-induced cardiac hypertrophy.

### TR3 upregulates mTORC1 activity

**Figure 3:**
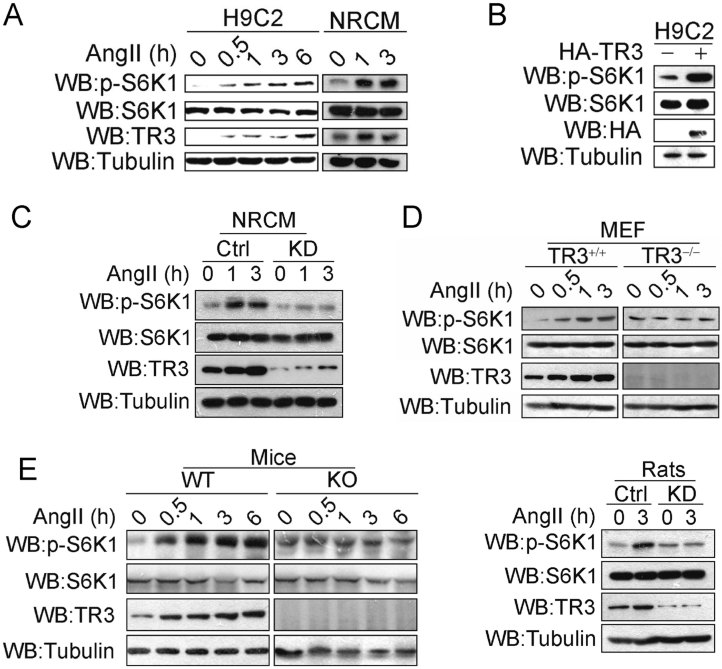
TR3 promoted mTORC1 activity Correlation between TR3 expression and S6K1 phosphorylation after AngII treatment of H9C2 cells and NRCMs. Cells were treated with 500 nM AngII for the indicated durations. TR3 expression and S6K1 activity were measured by Western blotting using TR3 and phosphor-Thr389-S6K1 antibodies.Transfection of TR3 into H9C2 cells increases the phosphorylation of S6K1. The transfected cells were lysed, and the lysates were analysed using Western blotting. An empty vector was used as a control.Knockdown of TR3 decreases mTORC1 activity even in the presence of AngII. Endogenous TR3 in the NRCMs was knocked down using lentivirus-based RNA interference. NRCMs that were transfected with scrambled siRNA were used as a control. The cells were treated with 500 nM AngII for the indicated durations, and S6K1 phosphorylation was determined.TR3-deficient (TR3^−/−^) MEFs exhibited a lower response to AngII than wild-type (TR3^+/+^) MEFs. TR3^+/+^ and TR3^−/−^ MEFs were obtained from WT and TR3-KO mice. The treatment was similar to that described above.TR3-KO mice and TR3-KD rats are not sensitive to AngII stimulation. WT and TR3-KO mice (left) or WT and TR3-KD rats, in which TR3 was knocked down in the left ventricles (right), were intraperitoneally injected with 500 µg/kg AngII at the indicated times. Total protein was prepared from the left ventricles of the mice or rats (*n* = 5 per group) and then subjected to Western blotting. Scrambled siRNA was used as a control.

To further characterize the role of TR3, we determined whether AngII influences the transcriptional activity of TR3 in the cardiac myoblast cell line H9C2. Transfection of a reporter gene for NurRE (the response element of TR3) into cells revealed that TR3 transcriptional activity was not affected by AngII treatment (Supporting Information Fig S3A). To verify this finding, EMSA was performed; the result excluded the possibility that AngII affected endogenous TR3 targeting to DNA (Supporting Information Fig S3B). In addition, AngII elevated the expression of the TR3 protein but not the mRNA levels in H9C2 cells and neonatal rat cardiomyocytes (NRCMs) isolated from WT rats (Fig 3A and Supporting Information Fig S3C). This finding might have been the result of the extension of the half-life of TR3 by AngII because when cycloheximide (CHX) was used to block *in vivo* protein synthesis, the half-life (*i.e.*, the time required for the degradation of 50% of the protein) of TR3 was prolonged by AngII (Supporting Information Fig S3D). These findings do not support the possibility that TR3 functions as a transcription factor to regulate cardiac hypertrophy; rather, the results suggest that TR3 is likely to be involved in regulation via protein–protein interactions.

Various studies have demonstrated that the mTORC1 pathway plays a central role in controlling cell size and pathological and physiological hypertrophy through its functions in protein synthesis (Gao et al, 2006). We found that TR3 was involved in regulating the AngII-induced changes in cell size during cardiac hypertrophy (Fig 1D) and that AngII-induced cardiac fibrosis was attenuated in WT mice that were co-treated with the mTORC1 inhibitor rapamycin (Supporting Information Fig S3E). Therefore, we predicted that TR3 may regulate the mTORC1 pathway. Indeed, the transfection of the HA-TR3 plasmids into H9C2 cells elevated the activity of mTORC1, as measured by the phosphorylation of S6K1 (Fig 3B). Moreover, we observed a strong correlation between TR3 expression and S6K1 phosphorylation when H9C2 cells were treated with AngII for different intervals (Fig 3A). To further assess the relationship between TR3 and mTORC1 activity, we isolated primary mouse embryonic fibroblasts (MEFs) from WT and TR3-KO mice in addition to NRCMs from WT rats. In the NRCMs, endogenous TR3 was effectively knocked down using a lentivirus-based RNA interference technique (termed TR3-KD; Supporting Information Fig S3F). AngII effectively enhanced the phosphorylation of S6K1 in the NRCMs (Fig 3A and C) and the WT (TR3^+/+^) MEFs (Fig 3D) but had no effect on the activity of mTORC1 in the TR3-KD NRCMs and the TR3-deficient (TR3^−/−^) MEFs. Similarly, we observed AngII-induced S6K1 phosphorylation in lysates extracted from the hearts of WT mice and left ventricles of WT rats but not in lysates extracted from the hearts of TR3-KO mice and left ventricles of TR3-KD rats (Fig 3E). Moreover, AngII similarly up-regulated TR3 protein expression, even in rapamycin-pretreated H9C2 cells (Supporting Information Fig S3G), which implies that this induction of TR3 by AngII occurs in the upstream of mTORC1 activation. Therefore, we conclude that TR3 participates in AngII-induced mTORC1 activity.

### TR3 interacts with the TSC1/TSC2 complex to regulate mTORC1 activity

**Figure 4:**
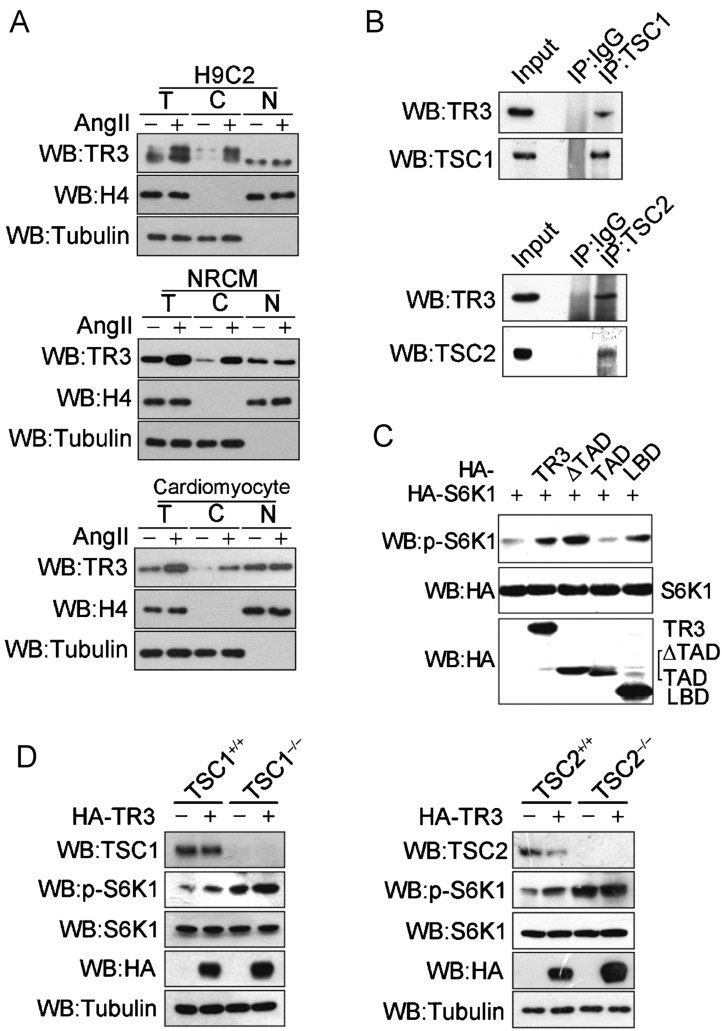
The interaction of TR3 with TSC1/TSC2 affects mTORC1 activity Cytoplasmic TR3 is upregulated after AngII stimulation in H9C2 cells (top), NRCMs (middle) and freshly isolated murine cardiomyocytes (bottom). The cells were treated with 500 nM AngII for 3 h. After the preparation of total (T), nuclear (N) and cytoplasmic (C) extracts, the expression of TR3 was determined by Western blotting. Histone 4 (H4) was used as a nuclear loading control, and tubulin was used as total and cytoplasmic loading controls.TR3 interacts with either TSC1 or TSC2 in the cytoplasmic fraction of mouse hearts (*n* = 3 per group). Heart cytoplasmic fractions were immunoprecipitated with a TSC1 (top) or TSC2 (bottom) antibody; the precipitates were then used to detect TR3 by Western blotting. IgG was used as a negative control.The interaction of TR3 with the TSCs is sufficient to influence the activity of mTORC1. HA-TR3 and its deletion mutants were introduced singly into 293T cells, and the phosphorylation of S6K1 was determined by Western blotting.The effects of TSC1 and TSC2 on TR3-induced mTORC1 activity. HA-TR3 was transfected into TSC1^+/+^ and TSC1^−/−^ MEF cells (left) or TSC2^+/+^ and TSC2^−/−^ MEF cells (right), and S6K1 phosphorylation was determined by Western blotting.

Because the stimulation of mTOR signalling occurs mainly in the cytoplasm and because our study shows that the regulation of mTORC1 activity by AngII is not associated with transcriptional activation by TR3, we postulated that cytoplasmic rather than nuclear TR3 may mediate AngII-induced mTORC1 activity. We analysed the distribution of TR3 in the nuclei and cytoplasms of H9C2 cells, NRCMs isolated from WT rats and fresh cardiomyocytes isolated from adult WT mice. Although TR3 was present in the nuclei and the cytoplasms, the AngII-induced expression of TR3 occurred only in the cytoplasms of these three cell types (Fig 4A). Immunohistochemical analysis verified the presence of cytoplasmic TR3 in normal and hypertrophied human heart tissue (Supporting Information Fig S4A), and AngII clearly induced cytoplasmic TR3 expression in the hearts of the WT mice (Supporting Information Fig S1B). These results suggest a possible function for TR3 in the cytoplasm.

The mechanism by which cytoplasmic TR3 regulates mTORC1 signalling is unknown. To determine whether TR3 interacts with specific partners that are required for the mTORC1 pathway, co-immunoprecipitation (co-IP) assays were conducted using mTOR, Rheb, Raptor, TSC1 and TSC2. In transfected cells, no interactions were detected between TR3 and mTOR, Rheb or Raptor (data not shown). However, when HA-TSC2 or Myc-TSC1 was precipitated using anti-HA or anti-Myc antibodies, respectively, Flag-TR3 was observed in the co-precipitated fraction. The reciprocal precipitation also demonstrated that TR3 was associated with TSC1 and TSC2 (Supporting Information Fig S4B). Furthermore, in the isolated cytoplasmic fraction of the mouse heart tissue, the precipitation of TSC2 and TSC1 using the respective antibodies demonstrated that endogenous TR3 was present in the TSC2 and TSC1 immunoprecipitations (Fig 4B). Immunofluorescent staining confirmed that TR3 was colocalized with TSC1 and TSC2 (Supporting Information Fig S4C). These results support the conclusion that TR3 interacts with TSCs.

TSC1 and TSC2 usually function as a dimer to negatively regulate mTORC1 activity. We used sequential immunoprecipitation and Western blotting to determine whether TR3 interacts with the TSC1/TSC2 complex to form a trimer. After cotransfecting plasmids encoding these proteins (Flag-TR3, Myc-TSC1 and HA-TSC2) into HEK293T cells, the lysates were precipitated using anti-Myc. Subsequently, the precipitates were eluted with Myc peptides and subjected to a second round of immunoprecipitation using an anti-Flag antibody. The final precipitation was analysed by Western blotting. The components obtained from the second round of immunoprecipitation included TR3, TSC1 and TSC2 (Supporting Information Fig S4D), which indicates that these three proteins formed a trimer.

To determine which region of the TR3 molecule is required for its interaction with TSC1 and TSC2, full-length TR3 and various deletion mutants were transfected into 293T cells, and immunoprecipitation assays were performed. TSC1 and TSC2 both bound to the ligand-binding domain (LBD) of TR3 (Supporting Information Fig S4E). We then investigated whether these interactions were sufficient for the upregulation of mTORC1 activity by TR3. Similar to TR3, the LBD and the ΔTAD constructs, which share the capacity to interact with the TSC complex, increased S6K1 phosphorylation. However, the TAD construct, which does not interact with the TSC1/TSC2 complex, had no effect on S6K1 phosphorylation (Fig 4C). Therefore, the TSC1/TSC2 complex may provide a platform for the stimulation of the TORC1 activity by TR3. TR3 lost its capacity to enhance the phosphorylation of S6K1 in TSC1-deficient and TSC2-null MEFs (Fig 4D), which further suggests the existence of a novel cross-talk mechanism between TR3 and the TSC1/TSC2 complex in the mTORC1 signalling pathway.

### TR3 promotes the degradation of TSC2 via the ubiquitination pathway

**Figure 5:**
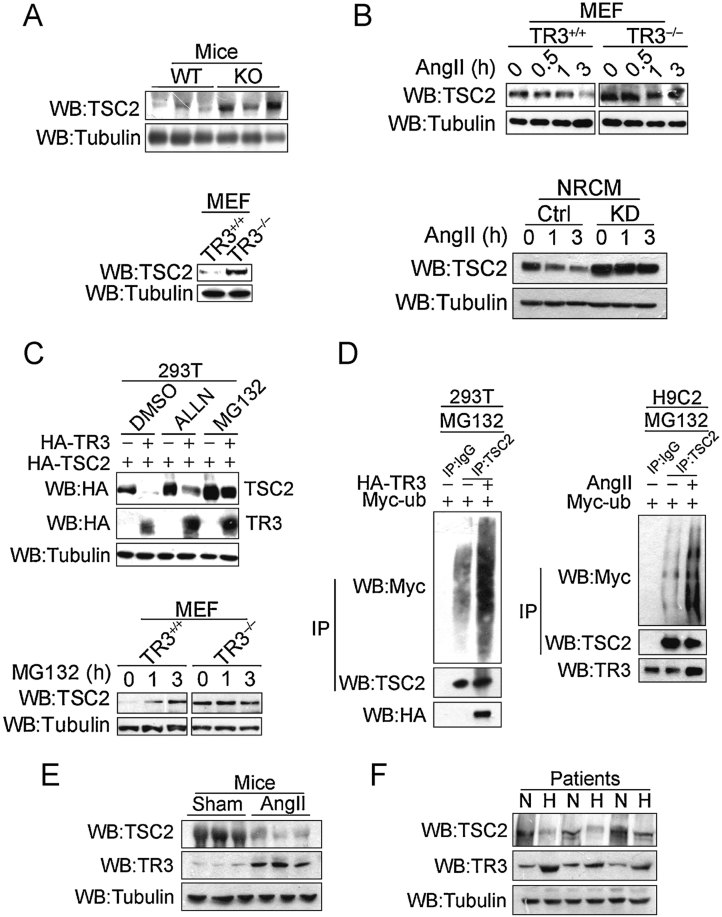
TSC2, but not TSC1, is degraded by TR3 through a proteasome/ubiquitination pathway Top, the expression of TSC2 is higher in TR3-KO mice than in WT mice. Hearts dissected from three individual mice were lysed, and TSC2 was detected by Western blotting. Bottom, TR3^−/−^ MEFs expressed higher levels of TSC2 than WT (TR3^+/+^) MEFs.AngII promotes the degradation of endogenous TSC2 in MEFs and NRCMs. The cells were treated with 500 nM AngII for the indicated durations and then lysed for the measurement of TSC2 protein by Western blotting.TR3 downregulates TSC2 via the proteasomal pathway. Top, HA-TR3 and HA-TSC2 were cotransfected into 293T cells; before harvesting, the cells were treated with 40 µM ALLN or 10 µM MG132 for 3 h. DMSO was used as a control. TSC2 protein levels were assessed by Western blotting. Bottom, MG132 stabilizes TSC2 protein levels in the MEFs but not the TR3^−/−^ MEFs. The cells were treated with 10 µM MG132 for the indicated durations and then harvested for Western blotting analysis.TR3 (left) or AngII (right) facilitates the recruitment of ubiquitin to TSC2. Myc-ubiquitin was introduced with TR3 into 293T (left) or H9C2 (right) cells. The cells were pretreated with 10 µM MG132 for 3 h and subsequently treated with 500 nM AngII for 3 h. TSC2 was immunoprecipitated, and a Myc antibody was used to detect ubiquitin-conjugated TSC2.AngII-treated mice exhibited higher TR3 and lower TSC2 expression than the sham-treated animals. Male mice (*n* = 3) were euthanized, and total protein was extracted for Western blotting.Patients with left ventricular hypertrophy exhibit higher TR3 and lower TSC2 expression. Protein lysates extracted from the left ventricular heart tissue of patients were immunoblotted with TR3 and TSC2 antibodies. Normal (N) and hypertrophic (H) hearts were determined as described in the Materials and Methods section.

Unexpectedly, we found that the endogenous and exogenous expression of TSC2 but not TSC1 decreased when TR3 was introduced into various cell types (Fig 4D and Supporting Information Fig S5A). TR3-KO mice consistently expressed TSC2 at much higher levels than WT mice (Fig 5A, top); this was also observed when comparing TR3^−/−^ MEFs with TR3^+/+^ MEFs (Fig 5A, bottom). This decrease of TSC2 by AngII was attenuated in TR3^−/−^ MEFs and TR3-KD NRCMs (Fig 5B). Similarly, CHX significantly decreased TSC2 expression in TR3^+/+^ MEFs but not in TR3^−/−^ MEFs (Supporting Information Fig S5B), which indicates that TSC2 is more stable in TR3^−/−^ MEFs than in TR3^+/+^ MEFs. These results strongly suggest a possible role for TR3 in the degradation of TSC2.

The N-terminal (1-418) region of TSC2, to which TSC1 binds, is essential for the stability of TSC2. We mapped the region of TSC2 that is responsible for the TR3 interaction by transfecting full-length TR3 and TSC2 deletion constructs into 293T cells. Co-IP assays showed that TR3 interacted with the D1 (1-418) and D2 regions of TSC2 but not with the D3 region, which contains a GAP domain (Supporting Information Fig S5C). Because the D1 region (1-418) is important for TSC2 proteasomal degradation, we determined whether TR3 influences the stability of TSC2 via the proteasomal pathway. MG132 and ALLN, which inhibit proteasome activity, effectively maintained TSC2 expression in the presence of TR3 (Fig 5C, top). In addition, the treatment of TR3^−/−^ MEFs with MG132 had no effect on TSC2 protein levels, whereas TSC2 protein levels were greatly increased by MG132 treatment of TR3^+/+^ MEFs (Fig 5C, bottom). If TSC2 undergoes proteasomal degradation in the presence of TR3, more ubiquitin-conjugated TSC2 should be observed as TR3 expression increases. As expected, when Myc-ubiquitin was introduced into 293T cells, binding of ubiquitin to endogenous TSC2 was clearly observed, and ubiquitination was significantly enhanced upon transfection with TR3 (Fig 5D, left). Consistently, upon the AngII-induced activation of TR3 in H9C2 cells, more ubiquitin was recruited to the TSC2 molecule (Fig 5D, right), which supports the conclusion that TR3 triggers the degradation of TSC2 through the proteasome/ubiquitination pathway.

The role of TR3 in the degradation of TSC2 was further assessed in AngII-treated mice and in samples from human patients with cardiac hypertrophy. The AngII-treated cardiac hypertrophic mice had higher levels of TR3 and correspondingly lower levels of TSC2 than sham controls (Fig 5E). Based on the LVPWd values for the clinical samples, the patients were divided into a hypertrophic group (LVPWd > 11 mm) and a normal group (LVPWd < 11 mm). The patients with cardiac hypertrophy had significantly higher levels of TR3 expression and corresponding lower levels of TSC2 (Table 2 and Fig 5F), which suggests that TR3 promoted the degradation of TSC2 and that the consequent induction of mTORC1 activity may be an important cause of cardiac hypertrophy.

**Table 2:** Echocardiographic parameters of human patients and relative protein levels

	Normal group (*n* = 15)	Hypertrophic group (*n* = 15)
IVSd (mm)	8.42 ± 1.29	12.34 ± 1.16***
LVIDd (mm)	49.26 ± 12.62	50.89 ± 8.58
LVIDs (mm)	30.86 ± 8.45	32.62 ± 6.56
LVPWd (mm)	8.67 ± 1.41	12.21 ± 1.02***
EF (%)	65.56 ± 6.54	64.32 ± 6.93
FS (%)	33.76 ± 5.83	34.43 ± 4.87
Relative TR3 level	1.12 ± 0.52	2.76 ± 0.82**
Relative TSC2 level	0.82 ± 0.33	0.29 ± 0.32**

After Western blotting, the protein levels of TR3 and TSC2 were quantified using Photoshop software. The levels of IVSd and LVPWd and the expression level of TR3 were very significantly elevated in the hypertrophic group compared to the normal group; in contrast, the expression level of TSC2 was significantly decreased in hypertrophic group compared to the normal group (***p < 0.001; **p < 0.01).

### TR3 regulates functions that depend on mTORC1 activity

**Figure 6:**
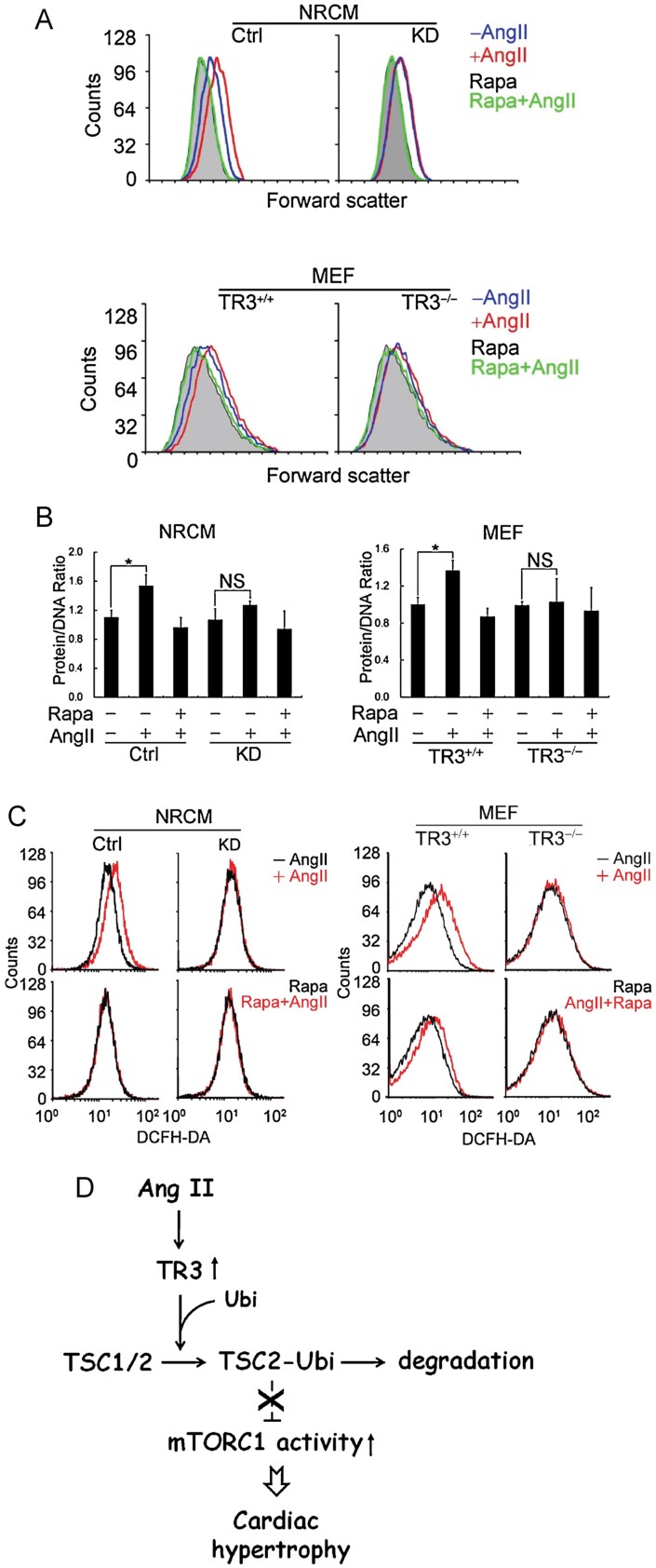
Regulation of cell size and ROS production by TR3 depends on mTORC1 signaling WT (TR3^+/+^) and TR3^−/−^ MEFs, and WT (Control) and TR3 knockdown (KD) NRCMs were obtained as described above. NRCM cells that were transfected with scrambled siRNA were used as a control. The AngII-induced increase in cell size requires TR3. Cells were co-treated with 500 nM AngII or 10 nM rapamycin as indicated for 48 h and then harvested to measure cell size using flow cytometry after PI staining.The AngII-induced increases in the total protein to total DNA ratio require TR3. Cells were pretreated with rapamycin for 1 h and subsequently treated with AngII for 3 h. After co-staining with FITC and PI, the cells were analyzed using flow cytometry.The AngII-induced production of ROS requires TR3. Cells were pretreated with rapamycin for 1 h and then treated with AngII for another 3 h. Before harvesting, the cells were loaded with DCFH-DA for 30 min and then analysed using flow cytometry. In each panel shown above, the bars represent the means ± SD of three independent experiments. *p < 0.05, NS: non-significant.A working model demonstrating how cardiac hypertrophy may be regulated by TR3 under the effect of AngII.

One of the essential and specific functions of mTORC1 is the regulation of cell size. To further evaluate the biological significance of TR3 in the mTORC1 pathway, we measured the sizes of various types of cells. Flow cytometry analysis revealed that after treatment with AngII, NRCMs and MEFs were much larger than control cells, whereas TR3-KD NRCMs and TR3^−/−^ MEFs did not exhibit any obvious change in size (Fig 6A). Immunofluorescent staining further confirmed this observation (Supporting Information Fig S6A). The ratio of total protein to DNA, which is another indicator of mTORC1 activity, was consistent with the change in cell diameter; the protein-to-DNA ratio increased more significantly in the NRCMs and MEFs than in the TR3-KD NRCMs and TR3^−/−^ MEFs following AngII administration (Fig 6B). However, when the cells were co-treated with rapamycin, AngII lost its capacity to increase cell size and promote protein synthesis (Fig 6A and B). The level of ROS was also measured as an indicator of mTORC1 activity (Blagosklonny, 2008). AngII enhanced ROS activity in NRCMs and MEFs, and the increase in ROS activity caused by AngII was partially reversed by rapamycin (Fig 6C). However, the activity of ROS was not altered in TR3-KD NRCMs or TR3^−/−^ MEFs in response to AngII. These findings suggest that TR3 regulates cellular functions that are dependent on mTORC1 activity.

Because mTORC2 also regulates cardiac hypertrophy (Chaanine & Hajjar, 2011), we tested whether TR3 participates in AngII-induced mTORC2 activation by monitoring AKT phosphorylation of Ser473. AngII effectively activated AKT phosphorylation even in the presence of rapamycin; deficiency or knockdown of TR3 in MEFs and NRCMs did not affect AngII-stimulated AKT activity. As a positive control, mTORC1 activity (as indicated by S6K1 phosphorylation) was silenced when the cells were co-treated with rapamycin or in AngII-induced TR3-KD NRCMs and TR3^−/−^ MEFs (Supporting Information Fig S6B). Together, these results demonstrate that TR3 participates in the AngII-regulated mTORC1 pathway but not in the mTORC2 pathway.

## DISCUSSION

Pathological cardiac hypertrophy is a cardiovascular disease that has been associated with mortality worldwide. Although many factors have been shown to regulate cardiac hypertrophy, mTORC1 is especially important in the progression of this disease because of its central role in regulating protein synthesis and cell size. Therefore, a detailed understanding of the regulatory mechanism of mTORC1 activity is important for developing effective treatments for cardiac hypertrophy. In this study, we provide the first evidence that the orphan nuclear receptor TR3 positively regulates mTOR signalling, and this regulation is amplified in mouse and rat models of AngII-induced cardiac hypertrophy. In WT mice and rats, pathological cardiac hypertrophy and the associated enhancement of mTORC1 activity were observed following chronic AngII stimulation, whereas TR3-KO mice and TR3-KD rats in which TR3 was specifically knocked down in the left ventricle demonstrated attenuated cardiac hypertrophy and diminished mTORC1 activity. Although AngII is generally considered to cause hypertension, BP was altered similarly in WT and TR3-KO mice and in WT and TR3-KD rats following the administration of AngII. Moreover, propranolol, which blocks hypertension, had no effect on AngII-induced TR3 expression. In contrast, losartan, an AngII receptor antagonist, significantly blocked AngII-induced TR3 expression. Together, these results suggest that AngII-induced cardiac hypertrophy is mediated by TR3, and the observed AngII-induced TR3 expression is not caused by the effect of hypertension but rather by AngII receptor-mediated signalling in cardiomyocytes. Further exploration of the mechanism underlying this effect revealed that the activation of mTORC1 by TR3 occurred through the interaction of TR3 with the TSC1/TSC2 complex. Through this interaction, TR3 promoted the ubiquitination and proteasomal degradation of TSC2 and subsequently activated mTORC1 (Fig 6D). Accordingly, disruption of the interaction between TR3 and the TSC may be a novel approach for the prevention of cardiac hypertrophy and may permit the development of a new class of therapeutic drugs for hypertrophic ablation.

As a transcription factor, TR3 primarily exerts its effects in the nucleus. However, in this study, we obtained unexpected evidence that AngII-induced TR3 functions mainly in the cytoplasm of cardiomyocytes and in the heart tissue of mice/rats. This finding is quite different from previously reported findings in which nuclear TR3 was shown to be recruited to its response element for steroid enzyme gene regulation following AngII treatment in H295R adrenal cells (Kelly et al, 2004). Because AngII has no impact on the transcriptional activity of TR3 in cardiomyocytes, the TR3-mediated effects of AngII may differ between various cells and organs. One possible explanation for this effect may be related to the distinct intracellular distribution of TR3. Recently, Cheng et al showed that although TR3 is primarily located in the nuclei of sham-operated hearts, a small amount of cytoplasmic TR3 is also detectable in the heart tissue, which is consistent with our findings (Cheng et al, 2011). However, in the report of Cheng et al, the expression level of TR3 was unchanged during ischemia-reperfusion (I/R) injury; rather, TR3 was translocated from the nucleus to the cytoplasm and finally to the mitochondria, where it initiated cell apoptosis via the induction of cytochrome c release, which supports the hypothesis that cytoplasmic TR3 may exert a regulatory function in heart tissue (Cheng et al, 2011). In our study, AngII elevated cytoplasmic TR3 levels but did not initiate the shuttling of TR3 to the mitochondria (data not shown). Rather, induction by AngII caused more TR3 to be targeted to the TSC complex, which resulted in the degradation of TSC2 through the recruitment of ubiquitin. As the result of this cascade, mTORC1 was activated, which led to increased protein synthesis and enlarged cell size; ultimately, cardiac hypertrophy was induced. It thus appears that the functions of TR3 and its regulatory signals are different in the I/R and AngII models.

Regulation of the TSC complex by protein–protein interactions has been reported. For example, 14-3-3β binds to TSC1 and TSC2 to form a ternary complex and ultimately attenuates the function of the TSC by changing its conformation (Shumway et al, 2003). Proteins associated with Myc (Pam) interact with the TSC to promote its degradation via an ubiquitin-mediated pathway (Murthy et al, 2004). FOXO1 disrupts the TSC1–TSC2 interaction via the competitive binding of TSC2 (Cao et al, 2006). These interactions are all accompanied by the activation of mTORC1. In this study, TR3 was shown to form a ternary complex with TSC1 and TSC2. Although TR3 interacts with the N-terminal region of TSC2 (1-418), which is the same domain to which TSC1 binds, the binding of TR3 did not perturb the interaction between TSC1 and TSC2 (unpublished observations). Rather, TR3 triggered TSC2 degradation by recruiting ubiquitin. Evidence from clinical and mouse samples has consistently demonstrated that TR3 is highly expressed and that TSC2 is weakly expressed in patients or mice with left ventricular hypertrophy. Furthermore, AngII enhanced the effect of TR3 on TSC2 degradation in mice.

**The paper explained**

PROBLEM:

Persistent cardiac hypertrophy results in various pathological changes, ultimately leading to cardiovascular disease. Although up-regulation of TR3, a member of the steroid/thyroid/retinoid receptor family, has been reported to occur during transverse aorta constriction-induced cardiac hypertrophy, the functional mechanism of TR3 in the development of AngII-induced cardiac hypertrophy is unknown.

RESULTS:

TR3 deficiency in mice or TR3 knockdown in the left ventricle of rats attenuates AngII-induced cardiac hypertrophy compared with the respective controls. A mechanistic analysis demonstrates that the TR3-mediated activation of mTORC1 is associated with AngII-induced cardiac hypertrophy. TR3 activates mTORC1 signalling by degrading TSC2 via the proteasomal ubiquitination pathway. TR3 expression was higher, whereas TSC2 expression was lower in clinical samples of hypertrophic patients.

IMPACT:

TR3 participates in AngII-induced cardiac hypertrophy by controlling mTORC1 signalling. As a nuclear receptor, TR3 is first reported to play an essential role in the development of AngII-induced cardiac hypertrophy. This study not only enriches our understanding of the molecular mechanisms of TR3 in the regulation of cardiac hypertrophy but also provides a novel target for the clinical therapy of cardiac hypertrophy.

In summary, we have shown that TR3 plays an essential role in the cardiac hypertrophy caused by the chronic elevation of AngII. This study reveals the existence of cross-talk mechanisms between TR3 and the TSC that function in the regulation of mTOR signalling and demonstrates a novel physiological function for TR3 that may qualify it as a clinical target for the treatment of cardiac hypertrophy.

## MATERIALS AND METHODS

### Cell culture and transfection

The immortalized rat cardiomyocyte cell line H9C2 was purchased from the Institute of Cell Biology, Shanghai, China. The human embryonic kidney 293T cell line was obtained from the American Type Culture Collection (ATCC, Maryland). TSC1-knockout (TSC1^−/−^) and TSC2-knockout (TSC2^−/−^) MEF cell lines were a gift from Professor Hong-bing Zhang (Peking Union Medical College, Beijing, China). Primary MEFs were isolated from 13.5-day-old mouse embryos, and primary NRCMs were isolated from the hearts of 3-day-old rats using standard procedures. All of the cells were cultured in Dulbecco's modified Eagle medium (DMEM) supplemented with 10% fetal bovine serum, 100 U/mL penicillin and 100 U/mL streptomycin. The 293T cells were transfected using the calcium phosphate precipitation method, and a TurboFect kit (MBI Fermentas) was used to transfect the other cell lines. All cells were serum-deprived for 12 h before AngII treatment.

### Animal experimental protocols

All animal experiments were approved by the Animal Ethics Committee of Xiamen University (Accept No.: XMULAC20120030).

Wild-type and TR3-KO mice on the C57BL/6J background were purchased from the Jackson Laboratory (Bar Harbor, Maine, USA). The specific knockdown of TR3 in the left ventricles of the rats was established as described in the following section. All of the animals were housed in the Xiamen University Laboratory Animal Center (Xiamen University, Fujian, China) under institutional guidelines. Male wild-type mice and TR3-KO littermates (8–12 weeks, 24–28 g) and rats (200–250 g) were used in the experiments.

#### Short-term administration of AngII

The mice or rats were intraperitoneally injected with 500 µg/kg of AngII. Control animals received a similar volume of 0.9% saline. The animals were euthanized at various time points, and the hearts were dissected and stored in liquid nitrogen for further study.

#### The establishment of AngII-induced cardiac hypertrophy in mice

AngII-induced cardiac hypertrophy was established in the mice according to a previously described method (Hu et al, 2004). Briefly, age- and weight-matched mice were subcutaneously implanted with osmotic minipumps (Alzet 1004, DURECT Corporation, Cupertino, CA). The sham group received 0.9% saline with acetic acid (0.01 M) over the course of the experiment, whereas AngII (Sigma) was constantly administered to the experimental group at a rate of 0.5 µg/kg/min for 4 weeks. BP was monitored using a Softron Indirect Blood Pressure Meter (BP98A, Softron).

Rapamycin (Sigma) or its vehicle was intraperitoneally administered to sham-operated or AngII-injected wild-type mice at the dose of 2 mg/kg/d for 4 weeks as previously described (Shioi, 2003). Rapamycin was dissolved in a solution of 0.2% sodium carboxymethylcellulose and 0.25% polysorbate-80 in water.

#### The treatment of mice with losartan and propranolol

One day after the establishment of cardiac hypertrophy, the mice were given 200 mg/L losartan (Sigma) or 100 mg/L propranolol (Sigma) in their drinking water for 2 weeks.

#### AngII-induced cardiac hypertrophy in the WT and TR3-KD rats

The rats were anaesthetized with 60 mg/kg pentobarbital sodium, and the thoracic cavity was accessed based on a previously described method (Belke et al, 2006). Briefly, the rats were ventilated with ambient air, and the chests were opened for viral injection. In the WT rats, viruses containing scrambled RNA (control group) were directly injected into the left ventricle using an insulin syringe. The TR3-KD rats were injected with virally expressed siTR3 in the left ventricle (siTR3: 5′-GCATTATGGTGCCGCACATT-3′). After the release of any trapped air, the rats were sutured and allowed to recover. Subsequently, AngII (0.5 µg/kg/min) was injected continuously into the WT and TR3-KD rats using osmotic minipumps (Alzet 1002, DURECT Corporation, Cupertino, CA) to induce cardiac hypertrophy. BP was monitored using a tail-cuff method (Model 29 Recorder and Model 179 Amplifier, IITC Life Science Inc.).

#### Echocardiography

Echocardiographic analyses were conducted on the anaesthetized mice or rats before and after the operation using a GE Medical Systems Vivid 7 Dimension (GE Healthcare) equipped with a 14-MHz animal application probe (i13L). The IVSd dimension and the LVPWd dimension were measured as previously reported (Schultz et al, 1999).

### Heart weight measurement

The hearts were dissected from the mice, washed in PBS to remove any blood and stored at 60°C for 48 h to remove the water.

### Human heart tissues

All clinical tissues were collected via standardized operative procedures approved by the Ethical Board of Xiamen Heart Center, Zhongshan Hospital, Xiamen University, Xiamen, Fujian Province, China, and conformed to the principles set out in the WMA Declaration of Helsinki and the NIH Belmont Report. Informed consent was obtained for all tissue samples linked with clinical data.

Patients who had underwent aortic valve replacement (AVR) surgery were divided into two groups based on the LVPWd values as assessed by quantitative echocardiography. The hypertrophic group exhibited LVPWd values >11 mm, and the normal group exhibited LVPWd values <11 mm. During surgery, myectomy samples were removed from the LV septum and immediately frozen in liquid nitrogen.

### Statistics

All values are reported as the means ± SD, and all comparisons were analysed using a *t-*test or a one-way ANOVA followed by a *t*-test.

### Other methods

The details of the other methods used, including Western blotting, co-immunoprecipitation assays, nuclear and cytosol fractionation, EMSA, immunofluorescent staining, flow cytometry, real-time PCR and the isolation of NRCMs and adult mice cardiomyocytes are provided as Supporting Information.

## Supplementary material

Detailed facts of importance to specialist readers are published as ”Supporting Information”. Such documents are peer-reviewed, but not copy-edited or typeset. They are made available as submitted by the authors.
