# Supplementary material for: The orphan receptor TR3 participates in angiotensin II-induced cardiac hypertrophy by controlling mTOR signalling
Source: EMBO Mol Med. 2012 Nov 29;5(1):137–48. doi: 10.1002/emmm.201201369 (PMC3569659; doi:10.1002/emmm.201201369)
Supplement: Supplementary file 1 [file emmm0005-0137-SD1.pdf]

EMBO Molecular Medicine

Manuscript EMM-2012-01369

The orphan receptor TR3 participates in angiotensin II-induced cardiac hypertrophy by controlling mTOR signaling

Rong-Hao Wang, Jian-Ping He, Mao-Long Su, Jie Luo, Ming Xu, Xiao-Dan Du, Hang-Zi Chen, Wei-Jia Wang, Yuan Wang, Nan Zhang, Bi-Xing Zhao, Wen-Xiu Zhao, Zhong-Gui Shan, Jia-Huai Han, Chawnshang Chang, and Qiao Wu

Corresponding author: Qiao Wu, Xiamen University

Review timeline:

Submission date:	13 March 2012
Editorial Decision:	16 April 2012
Revision received:	26 July 2012
Editorial Decision:	10 September 2012
Revision received:	08 October 2012
Accepted:	10 October 2012

Transaction Report:

(Note: With the exception of the correction of typographical or spelling errors that could be a source of ambiguity, letters and reports are not edited. The original formatting of letters and referee reports may not be reflected in this compilation.)

1st Editorial Decision 16 April 2012

Thank you for the submission of your manuscript "The orphan receptor TR3 participates in angiotensin II-induced cardiac hypertrophy by controlling mTOR signaling" to EMBO Molecular Medicine and please accept my apologies for the delayed reply. We have now received the reports from the three referees whom we asked to review your manuscript. You will see that they find the topic of your manuscript potentially interesting. However, they also raise significant concerns on the study, which should be addressed in a major revision of the manuscript.

Importantly, the reviewers highlight that the suggested molecular mechanism should be more convincingly demonstrated in the animal models and that different cell types should be individually analyzed. Reviewers #1 and #2 both note that the role of TR3 in pressure overload-induced hypertrophy should be further analyzed. In addition, all of the reviewers raise a number of technical concerns that should be convincingly addressed.

Given the balance of these evaluations, we feel that we can consider a revision of your manuscript if you can convincingly address the issues that have been raised within the time constraints outlined below.

However, we realize that addressing all of the referees' criticisms might require a lot of additional time and effort and be technically challenging. I am uncertain whether you will be able (or willing) to return a revised manuscript within the 3 months deadline and I would also understand your decision if you chose to rather seek rapid publication elsewhere at this stage.

Should you decide to embark on such a revision, revised manuscripts should be submitted within three months of a request for revision. They will otherwise be treated as new submissions.

Should you find that the requested revisions are not feasible within the constraints outlined here and choose, therefore, to submit your paper elsewhere, we would welcome a message to this effect.

Yours sincerely,

Editor
EMBO Molecular Medicine

***** Reviewer's comments *****

Referee #1 (Comments on Novelty/Model System):

The authors present a novel regulatory mechanism of mTOR pathway by AII via TR3 orphan nuclear receptor. Evidence is presented that TR3 associates with TSC1/TSC2 complex, which targets TSC2 to ubiquitination/degradation, resulting in upregulation of downstream mTOR signaling complex. Functional studies both in cells and in animal models suggest that this mechanism may be a critical mediator of the hypertrophic and deleterious effects of AII on the heart. The data presented is clearly of high importance and well balanced with a combination of molecular and functional data.

One weak point, in the opinion of this reviewer, is on the validation of the molecular mechanisms on the animal models. There is not enough data provided to verify that the TR3-TSC1/TSC2 mechanism is critical for the hypertrophic and deleterious effects of AII in the heart. Moreover, the animal model data heavily relies on western blots of whole cardiac tissue (either total or fractions) and immunohistochemistry from samples of WT and TR3-KO mice, KD rat and human, which are not fully satisfactory because they cannot distinguish the cellular types.

Concerning the molecular mechanisms, it should be noted that the data presented in this manuscript do not fully support a direct interaction between TR3 and TSC1/TSC2 complex. Additionally, no data is provided on how TR3 is particularly activated in the cytosol to interact with TSC1/TSC2 complex, neither how the interaction with TR3 prompts TSC2 to ubiquitination. Clearly, identifying these exact mechanisms are beyond the scope of this manuscript, however, in its absence a thorough demonstration of the involvement of the TR3-TSC1/TSC2 in the in the animal models is even more important.

In summary, this reviewer feels that the main conclusion drawn on the experimental results is quite novel and interesting. However, several clarifications are needed and additional experiments are necessary.

Referee #1 (Other Remarks):

The authors present a novel regulatory mechanism of mTOR pathway by AII via TR3 orphan nuclear receptor. Evidence is presented that TR3 associates with TSC1/TSC2 complex, which targets TSC2 to ubiquitination/degradation, resulting in upregulation of downstream mTOR signaling complex. Functional studies both in cells and in animal models suggest that this mechanism may be a critical mediator of the hypertrophic and deleterious effects of AII on the heart. The data presented is clearly of high importance and well balanced with a combination of molecular and functional data.

One weak point, in the opinion of this reviewer, is on the validation of the molecular mechanisms on the animal models. There is not enough data provided to verify that the TR3-TSC1/TSC2 mechanism is critical for the hypertrophic and deleterious effects of AII in the heart. Moreover, the animal model data heavily relies on western blots of whole cardiac tissue (either total or fractions) and immunohistochemistry from samples of WT and TR3-KO mice, KD rat and human, which are not fully satisfactory because they cannot distinguish the cellular types.

Concerning the molecular mechanisms, it should be noted that the data presented in this manuscript do not fully support a direct interaction between TR3 and TSC1/TSC2 complex. Additionally, no data is provided on how TR3 is particularly activated in the cytosol to interact with TSC1/TSC2 complex, neither how the interaction with TR3 prompts TSC2 to ubiquitination. Clearly, identifying these exact mechanisms are beyond the scope of this manuscript, however, in its absence a thorough demonstration of the involvement of the TR3-TSC1/TSC2 in the in the animal models is even more important.

In summary, this reviewer feels that the main conclusion drawn on the experimental results is quite novel and interesting. However, several clarifications are needed and additional experiments are necessary.

Specific Comments

Introduction

Page 2, last paragraph - where it says "specific receptor" change to "receptors". Although the regulation by TR3 is probably most related to the AT1 receptor subtype (Losartan data), the other subtypes may also play a role in the effects of AII in cardiac cells.

Page 3, 1st paragraph - Either edit or delete the paragraph. There is plenty of evidence for the prohypertrophic and deleterious effects of AII in the heart that should be summarized instead of using an argument based on the acute pharmacological effects of AII in patients. The same in Page 3, 2nd paragraph where it says "When used clinically, AII can induce cardiac hypertrophy".

Results

Page 5, last paragraph (Figure 1B-D)- Additional echocardiographic parameters such as LV diastolic and systolic diameters and fractional shortening are needed to fully characterize the cardiac phenotypes of the various groups. Gravimetric data shown in Figure 1C seems to conflict with the echocardiographic data as the level of attenuation of AII-induced hypertrophy is not so clear. ANOVA should be used in the comparisons of the 4 groups shown in Figure. Plus, the authors should provide additional data such as body weight and the heart weight-to-tibial length index.

Page 6, First paragraph (Figure 1D and S1B)- A better characterization of cardiomyocyte phenotype and protein distribution would include data from freshly isolated cells. Plus, immunohistochemistry of the whole cardiac tissue is not sufficient to exclude the contribution of the other cardiac cell types.

Page 6, last paragraph - Data shown in Figure S1A and Figure 1E, indicate that although treatment with beta-blocker reduces blood pressure and attenuate the hypertrophy, it is not accompanied by any significant change in the cardiac expression of TR3. This seems to conflict with a role for TR3 in pressure overload-induced hypertrophy.

Page 7, 2nd and 3rd paragraphs - Although the quantification of cardiac fibrosis and apoptosis, and the indirect assessment of pulmonary congestion provide some base to infer cardiac dysfunction, parameters of cardiac hemodynamic are needed to confirm changes in cardiac function.

Page 8, 2nd paragraph - Data demonstrating the validation of cardiac knockdown (e.g. specificity and efficiency of the interfering RNA) as well as the extension of the myocardial silencing needs to be provided.

Page 9, 1st paragraph - Need a positive control with exogenous TR3 for the reporter gene assay. The authors may want to provide additional data based on gel shift assay to further exclude the possibility that AII is not enhancing the binding of endogenous TR3 on DNA.

Page 10, 2nd paragraph -TR3 interacts with the TSC1/TSC2 complex to regulate mTOR activity. Figure 4A and 4B - Subcellular distribution should be assessed in freshly isolated adult cardiomyocyte from WT and TR3-KO mice. Plus, the efficiency of subcellular fractionation should

be confirmed with anti-histone and anti-myosin antibodies. The fractional subcellular distribution of TR3 should be based on comparison with the expression levels in the whole lysates.

Referee #2 (Comments on Novelty/Model System):

This is one of the strangest papers that I have ever reviewed. It has taken me a whole day to review it. The major problem is that there are really two papers on different topics that have been amalgamated into one. This means that an immense amount of data has been placed in the Supplementary Figures section, and the data are placed in the Figures section and in the Supplementary Figures section essentially at random. The authors have investigated the roles of TR3 (Nur77, nr4a1) in 'cardiac hypertrophy'. I did not think that this part of the work was particularly convincing. They have also performed a much less applied investigation of the degradation of TSC2 that is apparently induced by TR3. To me, the evidence was more convincing, though I find it a little difficult to believe. This paper cannot be accepted in its present form and I think that the authors would be better advised to divide it into two papers. It is a pity that they have chosen to take the approach that they have done, because each of the sections is not without interest. However, the paper is not coherent in its present form and this precludes acceptance.

NOTE 1. It is difficult to assess 'the adequacy of the model system' because so many different systems have been used. Some systems were adequate,others were less so.

NOTE 2. I feel that there is just too much data in this submission for a single publication. The authors seem to want to place a large quantity of significant data in the Supplementary section. Readers are often somewhat reluctant to read the Supplementary section because it is not as easy access as the publication itself. Personally, I think that they would be better advised to submit two separate publications, one two a 'basic science' journal and one to a more medically-applied journal (EMBO Mol Med?).

Referee #2 (Other Remarks):

This paper suggests that TR3 deficiency attenuates 'cardiac hypertrophy'. Some less- applied experimentation indicates that TR3 enhces degradation of the TSC2 GTPase activating protein and this increase mTORC1-dependent signalling.

1. The standard of writing is such that a dramatic revision is essential. The Introduction is long, repetitive and portions are irrelevant. Furthermore, sections are out of date. For example, the authors refer to mTORC rather than mTORC1 and they do not mention initially that TSC is a dimer of TSC1 and TSC2. They never mention in the Introduction that TSC2 is a GTPase activating protein (GAP) and Rheb is mentioned only peripherally. Although the authors state that GTP-loaded Rheb interacts directly with mTOR, how Rheb.GTP activates mTORC1 is still not understood. The references to mTORC1 are antediluvian. The field has advanced rapidly in recent years.
2. Page 5 lines 10-16. These sentences are poorly expressed. Currently, the phrasing suggests that TR3 expression is up-regulated in the TR3-knockout.
3. Following on from point 2, it would have been useful to show the expression of 'TR3' in the TR3 knockout (the authors should have the samples). This would be a useful control to establish the specificity of the TR3 antibody. It may be that such data are included later in the MS, but it would have been useful to see them here.
4. Fig. 1A. The text on the Fig. should presumably read 'AngII (w)', not 'AngII (h)'.
5. Fig. 1C. After what duration of AngII infusion was the heart wt./body wt. measured? Presumably it was 4 weeks.
6. Fig. 1C. The authors have not made the correct statistical comparison. The comparison that should be made is whether the heart wt./body wt. ratios in the two line of AngII-infused mice (WT and KO) were significantly different. There is however, clearly a discrepancy here between the data in Fig. 1C and Fig. 1B, because the ventricular diminsions are clearly different following AngII infusion of the two lines.
7. Fig. 1D. These are very impressive sections. It is a pity that the authors did not use planimetry to

estimate myocyte cross-sectional areas. Even if average cross-sectional areas were not statistically different, it looks to me as if the distribution of cell size is altered with changed means or medians.
8. Figs. 1E/1F. There is an obvious paradox here. Propranolol blocks the effects of AngII on (systolic?) blood pressure (Fig. 1A). Propranolol does not block the effects of AngII on TR3 expression (Fig. 1E). However, propranolol does prevent the increases in IVSd and LVPWd (two criteria used to establish 'hypertrophy'). To my mind, this shows that increased expression of TR3 is not an essential determinant for the hypertrophic response. The authors' conclusion at the top of page 7 ('Therefore, it is likely that AngII directly induces TR3 expression through its own receptor and that AngII-induced hypertension does not correlate with the increase in TR3 expression even though it causes cardiac hypertrophy.), although correct, is not really relevant. The authors' argument is that 'Our results reveal that deficiency of TR3 in mice or knockdown of TR3 in rat attenuated AngII-induced cardiac hypertrophy compared to their respective controls.' (see the Abstract).
9. Fig. 2A. Again, the authors have not performed the appropriate statistical analysis. The correct analysis is between the wild-type/AngII-infused group and the TR3 knockout/AngII infused group.
10. Fig. 2B. The blue fibrotic area seem to be superimposed on top of the myocytes. I do not understand this. What one normally observes in such sections is increased extracellular matrix surrounding the myocytes. I do not understand how a 'rate' of fibrosis can be calculated. Rates include a denominator of time. The normal way to express the degree of fibrosis is as a proportion of cross-sectional area, but that is not possible here because of the superimposition of the areas of fibrosis.
11. Fig. 2C. The authors have finally made the correct statistical comparison. Thereafter, they return to making invalid comparisons.
12. Page 7, three lines up. Left-ventricular heart failure is associated with pulmonary congestion. Cardiac hypertrophy is not necessarily associated with pulmonary congestion.
13. Page 8 line 5. CK-MB is not 'secreted' from myocytes. It leaches out of damaged myocytes.
14. Fig. S2A. What happens to TR3 protein expression in wild type and TR3-knockdown following AngII infusion? At the moment, no evidence is presented to indicate that TR3 protein expression differs.
15. Top of page 8. 'To characterize the underlying mechanism by which TR3 participates in cardiac hypertrophy'. At the moment, the evidence that TR3 participates in 'hypertrophy' is equivocal.
16. Fig. S3C. The authors assert that AngII increases the half-life of TR3 protein. The quantification of the data does not support this as there is no statistical analysis.
17. Fig. S3D. Again, the pattern of fibrosis is unusual.
18. Fig. 3B. How do the authors know that the increase in S6K phosphorylation did not result from the transfection? There is no control with the empty vector.
19. Does rapamycin inhibit the induction of TR3 protein by AngII? If it were to do so, it would imply that TR3 induction lies downstream from mTORC1 activation.
20. Fig. S3E. These experiments show TR3 transcript expression. The authors have already suggested that TR3 protein half-life might be a factor. What happens to TR3 protein in these experiments? What happens in AngII-treated cells?
21. Fig. 4B. Why does cytoplasmic and nuclear TR3 apparently migrate differently?
22. Page 11, second paragraph. The experiments in Fig. S4B are poorly-described. The authors do not mention in the text that they are using HEK293T cells. The left-hand panels are negative and do not really need to be shown. The right-hand panels are the reciprocal immunoprecipitations for TSC1 and TSC2 with TR3. I must admit that I am a little perplexed as to why different tags for TR3 (FLAG-TR3 and Myc-TR3) were used for the TSC2 co-immunoprecipitation, whereas the TSC1 co-immunoprecipitation just used the FLAG-TR3.
23. Page 12. It is perhaps important to mention that the reason that phospho-S6K is increased in the TSC1-deficient and the TSC2-null MEFs is that TSC1/2 cannot now act as a GAP for Rheb.GTP. However, this means that the argument is slightly circular because, if there is nothing for TR3 to inhibit, then it cannot inhibit anything.
24. Page 13, lines 4-5. 'The inhibition of TSC2 by AngII was attenuated in the TR3-/- MEFs and the TR3-KD NRCMs (Fig. 5B).' This should read 'The increased loss of TSC2 induced by AngII was attenuated in the TR3-/- MEFs and the TR3-KD NRCMs (Fig. 5B).'.
25. Fig. 5B. Cycloheximide does not appear to have been included in these experiments, though they probably would more reliable if it had been.
26. Page 13 lines 11-13. 'This result supports the hypothesis that TR3 influences TSC2 stability probably via the proteasomal pathway.' I do not understand this assertion. The fact that TR3 interacts with the D1 and D2 regions of TSC2 and the fact that the D1 region of TSC2 is essentially

for its stability do not indicate that the proteasomal pathway is involved.
27. The authors should mention that TSC1 interacts with the 1-418 N-terminal region of TSC2 and stabilises TSC2.
28. Fig. S5C. The authors should indicate on the Fig. that they are blotting for TSC2(phospho-Ser1387), the site phosphorylated by AMPK.
29. Page 15 second paragraph. ROS are not an index of apoptosis, they are a likely cause of apoptosis.
30. Fig. 6A. I do not understand what the experiments with rapamycin are meant to show. My view is that rapamycin is simply reducing the sizes of the cells and this is independent of whether AngII is present or not. I suspect that rapamycin alone would reduce cell sizes and the authors should consider showing such an experiment.
31. Page 20 section 2. Presumably, the AngII was administered in 0.9% NaCl + 10 mM acetic acid? Note that acetic acid is not mentioned in the Legend to Fig. 1.
32. Some methods descriptions are absent. Page 31. I have not checked that all of the antibodies are mentioned, but two that definitely not mentioned are those against phospho-TSC2 and phospho-AMPK. Page 31, last paragraph. How was 'myc-ubiquitin introduced into cells'? The description of the cardiac myocyte preparation could be improved (differential attachment to what?). The coverslips used for the immunofluorescence were presumably coated?
33. Some Legends are distinctly substandard. Fig. S1 Legend. AngII infusion, not AngII implantation. Acetic acid is not mention in the AngII infusions. Tropomyosin antibody, not tropomycin. Fig. S3. The Nur77RE is spliced to a luciferase reporter, it is not just a Nur77RE. The authors claim to use cycloheximide at 100 mg/ml. This is over 0.3 M! Normally, cycloheximide is used at 20 micromolar for these types of experiments. There is no scale bar on Fig. S3D (the legend says that there is). Scale bars have the dimension of micrometres, not micromolar (see also elsewhere). Fig. S4. Myc peptide, not myc peptides (this also occurs elsewhere). Fig. S5. AngII does not induce AMPK or TSC2 phosphorylation, so TR3 cannot impair AngII-induced phosphorylation, because there is not any.
34. The nuclear fraction is going to be relatively impure. Normally, one would extract nuclear proteins from the low speed pellet with high NaCl. This was not done here

Referee #3 (Comments on Novelty/Model System):

This study was well performed. Especially, the novel finding on the interaction between TR3 and TSC is interesting. However, there are several concerns, including a conceptual concern on the mTOR signaling pathway. As recent papers demonstrated, mTOR forms two complexes: the rapamycin-sensitive complex mTORC1 and the rapamycin-insensitive complex mTORC2. Akt, a key downstream molecule of mTORC2, plays an important role in cardiac hypertrophy. In addition, previous reports showed that long-term rapamycin treatment inhibits Akt activation while in some cases, short-term rapamycin treatment enhances Akt activation (by inhibition of the negative feedback). Thus it is essential to examine Akt activation and evaluate the mTORC2 signaling as well as the mTORC1 signaling in this study.

Other concerns are in the in vivo studies using KO mice and rats infected with lentivirus carrying siTR3. They did not show the protein level of TR3. These should be fundamental data to demonstrate the quality of animal models in gene silencing. Especially in rats infected with lentivirus carrying siTR3, it is important to demonstrate the level of TR3 gene silencing in the heat because in general, viral infection using a direct injection generates gene transfer in limited area in the myocardium.

Referee #3 (Other Remarks):

Wang and colleagues investigated the role of TR3 in angiotensin (Ang) II-induced cardiac hypertrophy using TR3-KO mice, rats infected with lentivirus carrying siTR3, H9C2 cell, and neonatal rat cardiomyocytes. The authors observed that TR3 mediated Ang-II cardiac hypertrophy

via mTOR activation. They also demonstrated that TR3 interacted with TSC and accelerated the ubiquitination-mediated degradation of TSC2, resulting in activation of mTOR signaling. The study is interesting. The finding regarding the interaction between TR3 and the TSC/mTOR signaling is novel. However, there are several concerns.
1) mTOR forms two complexes: mTORC1 (rapamycin-sensitive) and mTORC2 (rapamycin-insensitive). In the manuscript, they did not examine or discuss anything about mTORC2 signaling. Akt, a downstream molecule of mTORC2, plays an important role in cardiac hypertrophy. In addition, previous reports demonstrated that long-term rapamycin treatment inhibits Akt activation. They should evaluate mTORC2 as well as mTORC1 activation.
2) In Figure 1, while they demonstrated expression level of TR3 in Ang-II-treated WT mice, there are no data to compare the protein levels of TR3 between WT and KO mice. This is also applied to the experiments with rats infected with lentivirus carrying siTR3 (Figure 3).
3) Although the authors pointed about tissue specificity for studying the signaling pathway in the heart, they did not confirm the cardiac-specific effect of siTR3 (they stated "organ-specific" in the text). They should show protein level of TR3 in the heart and other tissues to demonstrate the organ specificity in the system with lentiviruses.
4) In Figure 2D, since the results of Lung/Body weight look marginal, those do not indicate the beneficial effect of TR3 gene silencing on cardiac function in Ang II-induced cardiac hypertrophy. If they want to discuss about cardiac function, they should perform physiological assays such as echocardiography.
5) In Figure 5D, quantitative assay of cross section of cardiomyocytes should be performed with the tissue samples.
6) In Figure 1C, 2A, 2B, and 2D, a comparison between WT and KO should be important.
7) In Figure 2C, the authors should describe how many nuclei were used for the quantitative assay of TUNEL.
8) In Figure 6, to demonstrate the effect of rapamycin, Western blotting showing phosphorylation levels of S6K1 and Akt should be performed. This is applied to Supplementary Fig 3D too.
9) In Figure 6, representative photos of NRCM and MEF should be helpful to demonstrate the effect of TR3 in Agn II-induced hypertrophy. For demonstrating cardiomyocyte hypertrophy in NRCM, immunohistochemistry with sarcomeric proteins such as alpha-actinin is broadly used.
10) In Supplementary Fig 1B, it is not clear to observe localization and expression level of TR3. The authors should try immunohistochemical staining with different fluorescent colors for anti-tropomycyin and ant-TR3.
11) On page 15 (Figure 6C), they investigated the effect of TR2 in apoptosis. ROS is known to induce apoptosis (but not an index). If the authors want to investigate apoptotic change in the setting. They should study caspase signaling pathways. As mentioned above, Akt is a key molecule of the mTOR signaling pathway: the upstream molecule of mTORC1 signaling and the downstream molecule of mTORC2. It is well known that Akt inhibits apoptosis in cardiomyocytes. Thus they need to examine Akt activation as well if they discuss the effect of TR3 in Ang II-induced apoptosis.

1st Revision - authors' response 26 July 2012

Answer to the Reviewer 1

1. ***Page 2, last paragraph - where it says "specific receptor" change to "receptors". Although the regulation by TR3 is probably most related to the AT1 receptor subtype (Losartan data), the other subtypes may also play a role in the effects of AII in cardiac cells.*** We have rewritten “introduction”, this sentence did not appear in revised version.

2. ***Page 3, 1st paragraph - Either edit or delete the paragraph. There is plenty of evidence for the prohypertrophic and deleterious effects of AII in the heart that should be summarized instead of using an argument based on the acute pharmacological effects of AII in patients. The same in Page 3, 2nd paragraph where it says "When used clinically, AII can induce cardiac hypertrophy".***

We have reedited that paragraph in the section of “Introduction” (page 3).

3. ***Page 5, last paragraph (Figure 1B-D)- Additional echocardiographic parameters such as LV diastolic and systolic diameters and fractional shortening are needed to fully characterize the cardiac phenotypes of the various groups. Gravimetric data shown in Figure 1C seems to conflict with the echocardiographic data as the level of attenuation of AII-induced hypertrophy is not so clear. ANOVA should be used in the comparisons of the 4 groups shown in Figure. Plus, the authors should provide additional data such as body weight and the heart weight-to-tibial length index.***

We measured other parameters such as heart rate, blood pressure, LVIDd, LVIDs and FS in WT and TR3-KO mice. To indicate them clearly, we make a new table (Tab.1) to compare each parameter between WT and TR3-KO mice before and after AngII administration. The body weight/tibial length and heart weight/tibial length are also included in Tab.1. Meanwhile, we apply ANOVA statistical method for the comparisons. ANOVA statistical method is also described in Materials and Methods. (page 25)

As for the question "gravimetric data shown in Figure 1C seems to conflict with the echocardiographic data", it may be that we did this experiment using "wet" hearts, which may lead to error. This time, we use "dry" hearts and got a satisfying result (Fig. 1C). The method for "dry" heart measurement is described in Materials and Methods. (page 24)

4 ***Page 6, First paragraph (Figure 1D and S1B)- A better characterization of cardiomyocyte phenotype and protein distribution would include data from freshly isolated cells. Plus, immunohistochemistry of the whole cardiac tissue is not sufficient to exclude the contribution of the other cardiac cell types.***

This is very instructive to us. To isolate the cardiomyocytes from adult WT and TR3-KO mice, we got great help from Dr. Ming Xu (a new co-author in revised version). The results showed that AngII indeed enlarged the sizes of cardiomyocytes isolated from WT mice but not from TR3-KO mice (Fig. 1E). Furthermore, the distribution of TR3 protein in cardiomyocytes was determined by western blotting (Fig. 4A, bottom) and immunofluorescent staining (Supplementary Fig. S1B). These results indicated that TR3 localizes in both nucleus and cytoplasm, and AngII effectively enhance the protein level of TR3 in cytoplasm.

Based on the Fig. 4A in revised version, we don't think it is necessary to show the expression of TR3 in hearts, so we suggest delete the original Fig. 4A.

5. ***Page 6, last paragraph - Data shown in Figure S1A and Figure 1E, indicate that although treatment with beta-blocker reduces blood pressure and attenuate the hypertrophy, it is not accompanied by any significant change in the cardiac expression of TR3. This seems to conflict with a role for TR3 in pressure overload-induced hypertrophy.***

Although both losartan and propranolol can attenuate AngII-induced hypertrophy, they share different signaling pathways. Losartan, an antagonist to AngII receptor, can directly block the transduction of AngII signaling pathway through mediation of AngII receptor. The fact that the AngII-induced TR3 expression and cardiac hypertrophy were all blocked by losartan treatment indicates that AngII-induced TR3 expression is through AngII receptor, which is associated with cardiac hypertrophy. In contrast, propranolol is a beta-blocker, whose target is adrenergic receptor, rather than AngII receptor. Although treatment with propranolol reduces blood pressure and thus attenuates the hypertrophy, this drug seems not to directly interrupt the AngII signaling pathway in cardiomyocytes. Under this circumstance, the TR3 expression could still be stimulated by AngII. Since AngII-induced cardiac hypertrophy is a complicated process, it is conceivable that upregulation of TR3 by AngII in cardiomyocytes is required but not sufficient for cardiac hypertrophy. This viewpoint may also partially explain why WT mice do not show any cardiac hypertrophy when compared with TR3 KO mice in normal condition.

6. ***Page 7, 2nd and 3rd paragraphs - Although the quantification of cardiac fibrosis and apoptosis, and the indirect assessment of pulmonary congestion provide some base to infer***

cardiac dysfunction, parameters of cardiac hemodynamic are needed to confirm changes in cardiac function.

We completely agree that hemodynamic parameters such as dp/dt, which stands for the maximal rate of the change of intraventricular pressure, are determinants for cardiac function. However, to complete this experiment, a specific transducer to record dp/dt by cardiac catheterization is needed, which is beyond our reach. Nevertheless, fractional shortening (FS), which reflects cardiac contractional ability, could also determine the cardiac function [Mcmullen, J.R., et al. *Circulation*, 2004, 109(24): 3050-3055; Liao, Y., et al. *Circulation*, 2004, 110(6): 692-9; Frank, D., et al. *Circulation*, 2007, 116(22): 2587-96; Loyer, X., et al. *Circulation*, 2008, 117(25): 3187-98], without the use of this specific transducer. Therefore, FS is detected instead of hemodynamic study in revised version. As shown in Tab. 1, after AngII administration, FS decreased from 65.89% to 31.46% in WT mice, however, in TR3 KO mice, FS showed comparable levels before and after AngII administration (67.89% *vs* 62.42%).

These changes in detection have been agreed by you in advance. Thank you for your understanding.

7. ***Page 8, 2nd paragraph - Data demonstrating the validation of cardiac knockdown (e.g. specificity and efficiency of the interfering RNA) as well as the extension of the myocardial silencing needs to be provided.***

To indicate the efficiency and specificity of the interfering RNA in the left ventricles of rats, the right ventricles, liver, muscle and kidney from rats were isolated respectively to detect TR3 protein levels. The result show that the expression of TR3 in the left ventricles of rats was successfully knocked down, however, in other organs especially in the right ventricle, the TR3 levels was unchangeable. This effect of TR3 knockdown was maintained at least for two weeks in our case (Supplementary Fig. S2A).

8. ***Page 9, 1st paragraph - Need a positive control with exogenous TR3 for the reporter gene assay. The authors may want to provide additional data based on gel shift assay to further exclude the possibility that AII is not enhancing the binding of endogenous TR3 on DNA.***

According to your suggestion, we repeated reporter gene assay using exogenous TR3 as a positive control (Supplementary Fig. S3A). We also performed a gel shift assay to exclude AngII's effect on endogenous TR3 targeting to DNA. As expected, AngII did not affect endogenous TR3 binding to DNA, although a little bit of TR3 bound to DNA (Supplementary Fig. S3B).

9 ***Page 10, 2nd paragraph -TR3 interacts with the TSC1/TSC2 complex to regulate mTOR activity. Figure 4A and 4B - Subcellular distribution should be assessed in freshly isolated adult cardiomyocyte from WT and TR3-KO mice. Plus, the efficiency of subcellular fractionation should be confirmed with anti-histone and anti-myosin antibodies. The fractional subcellular distribution of TR3 should be based on comparison with the expression levels in the whole lysates.***

We isolated cardiomyocytes from adult WT and KO mice with the help of Dr. Ming Xu, and displayed subcellular distribution of TR3 (Fig. 4A, bottom). For detail, please refer to our answer in Question 4 above. According to your suggestion, we used anti-histone 4 (H4) to label the total and the nuclear fractions (Fig. 4). In addition, we ran another gel plus the total cell lysates. The result showed that AngII indeed induced TR3 expression in total cell lysates and cytoplasmic fractions in different cell lines (Fig. 4A).

Answer to the Reviewer 2

1. The standard of writing is such that a dramatic revision is essential. The Introduction is long, repetitive and portions are irrelevant. Furthermore, sections are out of date. For example, the authors refer to mTORC rather than mTORC1 and they do not mention initially that TSC is a

dimer of TSC1 and TSC2. They never mention in the Introduction that TSC2 is a GTPase activating protein (GAP) and Rheb is mentioned only peripherally. Although the authors state that GTP-loaded Rheb interacts directly with mTOR, how Rheb.GTP activates mTORC1 is still not understood. The references to mTORC1 are antediluvian. The field has advanced rapidly in recent years.

We have rewritten the section of Introduction according to your suggestion. (page 3-4)

2.Page 5 lines 10-16. These sentences are poorly expressed. Currently, the phrasing suggests that TR3 expression is up-regulated in the TR3-knockout.

We rewrote these sentences as " We first established a model of AngII-induced cardiac hypertrophy in which 12-week-old WT mice and age-matched TR3-knockout (TR3-KO) littermates were implanted with mini-osmotic pumps to administer AngII continuously for 4 weeks, and the TR3 expression in the WT mice and the blood pressure of mice with both genotypes were up-regulated within 4 weeks (Fig. 1A & Supplementary Fig. S1A). However, the blood pressure was comparable between the WT and TR3-KO mice, which suggests that a TR3 rather than a difference in blood pressure caused the different phenotype between the WT and TR3-KO mice." (page 6)

3. ***Following on from point 2, it would have been useful to show the expression of 'TR3' in the TR3 knockout (the authors should have the samples). This would be a useful control to establish the specificity of the TR3 antibody. It may be that such data are included later in the MS, but it would have been useful to see them here.***

The expression levels of TR3 protein in different cases are detected by western blotting□ including TR3-KD NRCMs (Supplementary Fig. S3F), TR3-KO MEFs (Fig. 3D), the left ventricle of TR3-KO mice (Supplementary Fig. S1A), and the left ventricle of TR3-KD rat, in which the TR3 expression level in the left ventricle was specifically knocked down (Supplementary Fig. S2A).

To show the specificity of TR3 antibody, we further detected TR3 expression in different organs, including the left ventricle, right ventricle, liver, muscle and kidney from WT and KD rats. The results show that the TR3 antibody we used has a good specificity and specifically knockdown of TR3 in the left ventricles of rats is successful (Supplementary Fig. S2A).

4. ***Fig. 1A. The text on the Fig. should presumably read 'AngII (w)', not 'AngII (h)'.***

We have revised this slip of the pen.

5. ***Fig. 1C. After what duration of AngII infusion was the heart wt./body wt. measured? Presumably it was 4 weeks.***

Yes, it is 4 weeks. We added this information in the Figure Legend (page 30).

6. ***Fig. 1C. The authors have not made the correct statistical comparison. The comparison that should be made is whether the heart wt./body wt. ratios in the two line of AngII-infused mice (WT and KO) were significantly different. There is however, clearly a discrepancy here between the data in Fig. 1C and Fig. 1B, because the ventricular diminsions are clearly different following AngII infusion of the two lines.***

According to the suggestion, we make statistical comparisons between AngII-infused WT and KO mice using the method of ANOVA. The results showed a significant difference between AngII-infused WT and KO mice (*, $p<0.05$, Fig. 1C).

The reviewer mentioned that there is a conflict between Fig.1C and Fig.1B. The reason may be that we did this experiment using "wet" hearts, which may lead to error. This time, we use "dry" hearts to do this experiment and got a satisfying result (Fig. 1C). The method of "dry" heart has been described (page 24).

7. ***Fig. 1D. These are very impressive sections. It is a pity that the authors did not use planimetry to estimate myocyte cross-sectional areas. Even if average cross-sectional areas were not statistically different, it looks to me as if the distribution of cell size is altered with changed means or medians.***

According to the suggestion, the mean cross-sectional areas of WT and TR3-KO cardiomyocytes were calculated using ImageJ software. Such calculation method is often used [Duan, S., et al. *Circ Res*, 2005, 97(4): 372-9; Kim, Y., et al. *J Clin Invest*, 2008, 118(1): 124–32]. As shown in Fig 1D, after AngII treatment, the mean cross-sectional area of WT cardiomyocytes significantly enlarged from 248.8 μm^2 to 412.7 μm^2, while the value of TR3-KO cardiomyocytes remained unchanging, indicating that TR3 involves in AngII-induced cardiac hypertrophy.

8. ***Figs. 1E/1F. There is an obvious paradox here. Propranolol blocks the effects of AngII on (systolic?) blood pressure (Fig. 1A). Propranolol does not block the effects of AngII on TR3 expression (Fig. 1E). However, propranolol does prevent the increases in IVSd and LVPWd (two criteria used to establish 'hypertrophy'). To my mind, this shows that increased expression of TR3 is not an essential determinant for the hypertrophic response. The authors' conclusion at the top of page 7 ('Therefore, it is likely that AngII directly induces TR3 expression through its own receptor and that AngII-induced hypertension does not correlate with the increase in TR3 expression even though it causes cardiac hypertrophy.), although correct, is not really relevant. The authors' argument is that 'Our results reveal that deficiency of TR3 in mice or knockdown of TR3 in rat attenuated AngII-induced cardiac hypertrophy compared to their respective controls.' (see the Abstract).***

Although both losartan and propranolol can attenuate AngII-induced hypertrophy, they share different signaling pathways. Losartan, an antagonist to AngII receptor, can directly block the transduction of AngII signaling pathway through mediation of AngII receptor. The fact that the AngII-induced TR3 expression and cardiac hypertrophy were all blocked by losartan treatment indicates that AngII-induced TR3 expression is through AngII receptor, which is associated with cardiac hypertrophy. In contrast, propranolol is a beta-blocker, whose target is adrenergic receptor, rather than AngII receptor. Although treatment with propranolol reduces blood pressure and thus attenuates the hypertrophy, this drug seems not to directly interrupt the AngII signaling pathway in cardiomyocytes. Under this circumstance, the TR3 expression could still be stimulated by AngII. Since AngII-induced cardiac hypertrophy is a complicated process, it is conceivable that upregulation of TR3 by AngII in cardiomyocytes is required but not sufficient for cardiac hypertrophy. This viewpoint may also partially explain why WT mice do not show any cardiac hypertrophy when compared with TR3 KO mice in normal condition.

We rewrote the conclusion "Therefore, it is likely that the upregulation of TR3 occurs directly through AngII signaling in cardiomyocytes but not through the AngII-induced hypertension". (page 8)

9. ***Fig. 2A. Again, the authors have not performed the appropriate statistical analysis. The correct analysis is between the wild-type/AngII-infused group and the TR3 knockout/AngII infused group.***

We have done it (Fig. 2A, *$p<0.05$).

10 ***The blue fibrotic area seem to be superimposed on top of the myocytes. I do not understand this. What one normally observes in such sections is increased extracellular matrix surrounding the myocytes. I do not understand how a 'rate' of fibrosis can be calculated. Rates include a denominator of time. The normal way to express the degree of fibrosis is as a proportion of cross-sectional area, but that is not possible here because of the superimposition of the areas of fibrosis.***

We repeated this experiment and got a satisfying result. (Fig.2B). By using imageJ software, we got the fibrotic proportion. In addition, we changed "fibrotic rate" to "fibrotic proportion".

11. ***Fig. 2C. The authors have finally made the correct statistical comparison. Thereafter, they return to making invalid comparisons.***

We have done (Fig. 1C, 2A, 2B).

12. ***Page 7, three lines up. Left-ventricular heart failure is associated with pulmonary congestion. Cardiac hypertrophy is not necessarily associated with pulmonary congestion.***

Yes, it has been reported that cardiac hypertrophy sometimes causes pulmonary congestion but there is no causal relationship between pulmonary congestion and cardiac hypertrophy [Kiss, E., et al. *Circ Res*. 1995, 77(4): 759-64; Jalili, T., et al. *Cardiovasc Res*, 1999; 44(1): 5-9]

In revised version, we used echocardiographic parameters to reflect heart function (Tab. 1). In addition, according to the suggestion from Review3#, we delete this data (original Fig. 2D).

13. ***Page 8 line 5. CK-MB is not 'secreted' from myocytes. It leaches out of damaged myocytes.***

Yes, you are right. After consideration, we deleted this data (orginal Fig. 2D). Instead, we performed echocardiography to reflect cardiac function (Tab. 1).

14. ***Fig. S2A. What happens to TR3 protein expression in wild type and TR3-knockdown following AngII infusion? At the moment, no evidence is presented to indicate that TR3 protein expression differs.***

After knockdown of TR3 in the left ventricles of rats or in NRCMs, AngII induced much weaker expression of TR3 than those in WT rats and NRCMs (Fig. 3C & 3E). In TR3-KO mice or TR3-KO ($TR3^{-/-}$) MEFs, TR3 expression could not be detected as expected (Fig. 3D & 3E).

15. ***Top of page 8. 'To characterize the underlying mechanism by which TR3 participates in cardiac hypertrophy'. At the moment, the evidence that TR3 participates in 'hypertrophy' is equivocal.***

We rewrote this sentence as “To characterize the role of TR3 further” (page 10).

16. ***Fig. S3C. The authors assert that AngII increases the half-life of TR3 protein. The quantification of the data does not support this as there is no statistical analysis.***

We repeated this experiment three times and then made a statistical analysis (Fig. S3D).

17. ***Fig. S3D. Again, the pattern of fibrosis is unusual.***

We repeated this experiment (Fig.S3E).

18. ***Fig. 3B. How do the authors know that the increase in S6K phosphorylation did not result from the transfection? There is no control with the empty vector.***

Actually, we used empty vector as a control for transfection in all of necessary experiments. We have described this in Figure legends (page 32).

19 ***Does rapamycin inhibit the induction of TR3 protein by AngII? If it were to do so, it would imply that TR3 induction lies downstream from mTORC1 activation.***

To answer this question, we pretreated H9C2 with 10 nM rapamycin for 1 hour, followed with AngII for 3 hours. The result showed that AngII similarly up-regulates TR3 expression even in the rapamycin-pretreated cells, indicating that TR3 induction does not lie downstream of mTORC1 activation (Supplementary Fig. S3G).

20 ***Fig. S3E. These experiments show TR3 transcript expression. The authors have already suggested that TR3 protein half-life might be a factor. What happens to TR3 protein in these experiments? What happens in AngII-treated cells?***

In response to this comment, the protein levels of TR3 were detected in control and TR3-KD NRCMs (Supplementary S3F). A much weaker induction of TR3 was observed in the AngII-treated TR3-KD NRCMs or TR3-KD rats in which the left ventricle of rat was specifically knocked down, as compared to the corresponding controls; however, this slightly increase of TR3 expression is not enough to affect the mTORC1 activity(Fig. 3C & 3E).

21 ***Fig. 4B. Why does cytoplasmic and nuclear TR3 apparently migrate differently?***

TR3 is a heavy phosphorylated protein [Fahrner, T.J, et al., *Mol Cell Biol*, 1990, 10(12): 6454-9]. The differential migration of TR3 in the nucleus and the cytoplasm may be due to the different phosphorylation status of TR3. It has been reported that phosphorylated modification promotes the nuclear exportation of TR3 [Fahrner, T.J., et al. *Mol Cell Biol*, 1990, 10(12): 6454-9; Katagiri, Y., et al. *Nat Cell Biol*, 2000, 2(7): 435-40; Han, Y.H., *et al. Oncogene*, 2006, 25(21): 2974-86], inferring that cytoplasmic TR3 may possess higher phosphorylation level. Therefore, cytoplasmic TR3 migrates slower than nuclear TR3 in western blotting. However, in NRCMs the cytoplasmic migration was not detected, suggesting different modification occurred in different cell lines.

22 ***Page 11, second paragraph. The experiments in Fig. S4B are poorly-described. The authors do not mention in the text that they are using HEK293T cells. The left-hand panels are negative and do not really need to be shown. The right-hand panels are the reciprocal immunoprecipitations for TSC1 and TSC2 with TR3. I must admit that I am a little perplexed as to why different tags for TR3 (FLAG-TR3 and Myc-TR3) were used for the TSC2 co-immunoprecipitation, whereas the TSC1 co-immunoprecipitation just used the FLAG-TR3.***

We rewrote the experiment of Fig. S4B in detail (page 13), and mentioned the cell line HEK293T in supplementary Figure legends (supplementary data page 12).

According to the suggestion, we delete left-hand panels of Fig. S4B, which showed no interactions between TR3 and Raptor, mTOR, or Rheb. To make a consistence in tag, we conducted Flag-TR3 plasmid. The co-IP assay showed an interaction between HA-TSC2 and Flag-TR3 (Fig. S4B), indicating there is no tag specificity.

23 ***Page 12. It is perhaps important to mention that the reason that phospho-S6K is increased in the TSC1-deficient and the TSC2-null MEFs is that TSC1/2 cannot now act as a GAP for Rheb.GTP. However, this means that the argument is slightly circular because, if there is nothing for TR3 to inhibit, then it cannot inhibit anything.***

We showed here that TR3 can no longer influence the phosphorylation level of S6K1 when TSC1 or TSC2 was knockout (Fig. 4D). This result helps us to exclude the possibility that TR3 might regulate mTOR activity through other signaling pathway. Therefore, the use of TSC1-KO and TSC2-KO MEFs in our study may further emphasize the TR3-TSC1/2-mTOR signaling transduction pathway.

24 ***Page 13, lines 4-5. 'The inhibition of TSC2 by AngII was attenuated in the TR3-/- MEFs and the TR3-KD NRCMs (Fig. 5B).' This should read 'The increased loss of TSC2 induced by AngII was attenuated in the TR3-/- MEFs and the TR3-KD NRCMs (Fig. 5B).'***

We have rewritten this sentence according to your suggestion (page 14).

25 ***Fig. 5B. Cycloheximide does not appear to have been included in these experiments, though they probably would more reliable if it had been.***

We detected the stability of TSC2 with the using of cycloheximide (CHX). As shown in Fig. S5B, CHX significantly decreased TSC2 protein level in TR3$^{+/+}$ MEFs but not in TR3$^{-/-}$ MEFs, indicating TSC2 is more stable in TR3$^{-/-}$ MEFs than TR3$^{+/+}$ MEFs. We repeated this experiment three times and made a statistic analysis.

26 ***Page 13 lines 11-13. 'This result supports the hypothesis that TR3 influences TSC2 stability probably via the proteasomal pathway.' I do not understand this assertion. The fact that TR3 interacts with the D1 and D2 regions of TSC2 and the fact that the D1 region of TSC2 is essentially for its stability do not indicate that the proteasomal pathway is involved.***

We have rewritten this sentence as "Because the D1 region (1-418) is important for TSC2 proteasomal degradation, we determined whether TR3 influences the TSC2 stability via the proteasomal pathway" (page 15).

27 ***The authors should mention that TSC1 interacts with the 1-418 N-terminal region of TSC2 and stabilises TSC2.***

We have mentioned that on page 15.

28 ***Fig. S5C. The authors should indicate on the Fig. that they are blotting for TSC2(phospho-Ser1387), the site phosphorylated by AMPK.***

As you mentioned below (question 33), AngII had no effect on AMPK activation in our case. We would like to delete this figure.

29 ***Page 15 second paragraph. ROS are not an index of apoptosis, they are a likely cause of apoptosis.***

Yes, ROS is a likely cause but not an index of apoptosis. On the other hand, ROS is also an indirect indicator to mTORC1 activity [Sharp, Z.D. et al., *Cell Mol Life Sci*, 2011, 68(4): 587-97; Lee, J.H., et al. *Science*, 2010, 327(5970): 1223-8]. Therefore, we changed this sentence from "we measured ROS activity, which is promoted by AngII partially through mTOR and is considered to be an index for apoptosis" to " ROS level is additionally measured as an indicator of mTORC1 activity (page 17).

30 ***Fig. 6A. I do not understand what the experiments with rapamycin are meant to show. My view is that rapamycin is simply reducing the sizes of the cells and this is independent of whether AngII is present or not. I suspect that rapamycin alone would reduce cell sizes and the authors should consider showing such an experiment.***

Treatment of rapamycin alone for 48 hours efficiently reduces cell size as compared with control group. Under this circumstance, AngII can no longer influence the cell size (Fig.6A & Supplementary Fig. 6A).

31 ***Page 20 section 2. Presumably, the AngII was administered in 0.9% NaCl + 10 mM acetic acid? Note that acetic acid is not mentioned in the Legend to Fig. 1.***

We have indicated them in the figure legend (page 29).

32 ***Some methods descriptions are absent. Page 31. I have not checked that all of the antibodies are mentioned, but two that definitely not mentioned are those against phospho-TSC2 and phospho-AMPK. Page 31, last paragraph. How was 'myc-ubiquitin introduced into cells'? The description of the cardiac myocyte preparation could be improved (differential attachment to what?). The coverslips used for the immunofluorescence were presumably coated?***

We have replenished some of information, including antibodies, ubiquitin transfection and cardiac myocyte preparation in Supplementary Methods (supplementary data page 2-3). The coverslips used for the immunofluorescence were coated with different reagents for different cells. Before immunofluorescence experiment, NRCMs were cultured on gelatin coated coverslips, and freshly isolated mice cardiomyocytes were cultured on laminin coated coverslips. The normal coverslips were used for H9C2 cells.

33 ***Some Legends are distinctly substandard. Fig. S1 Legend. AngII infusion, not AngII implantation. Acetic acid is not mention in the AngII infusions. Tropomyosin antibody, not tropomycin. Fig. S3. The Nur77RE is spliced to a luciferase reporter, it is not just a Nur77RE. The authors claim to use cycloheximide at 100 mg/ml. This is over 0.3 M! Normally, cycloheximide is used at 20 micromolar for these types of experiments. There is no scale bar on Fig. S3D (the legend says that there is). Scale bars have the dimension of micrometres, not micromolar (see also elsewhere). Fig. S4. Myc peptide, not myc peptides (this also occurs elsewhere). Fig. S5. AngII does not induce AMPK or TSC2 phosphorylation, so TR3 cannot impair AngII-induced phosphorylation, because there is not any.***

Thank you for your careful review, we have revised all slips of the pen.

The concentration of cycloheximide we used is 100 ug/ml. The scale bars is mm.

Fig.S5C was deleted because AngII had no effect on AMPK activation in our case.

34. The nuclear fraction is going to be relatively impure. Normally, one would extract nuclear proteins from the low speed pellet with high NaCl. This was not done here

There are different methods for preparation of nuclear fractions. The method we used is from Sussman's group [Cheng, Z., et al. *Eur Heart J*, 2011, 32(17): 2179-88], in which pure nuclear fraction can be obtained. In addition, tubulin is not detected in the nuclear fractions (Fig. 4A) further suggest that the nuclear fraction is pure enough and can be used in research.

In the revised version, we deleted the original Fig.4A, because review#1 requests us to isolate mice cardiomyocytes to exclude contributions of other cardiac cells. In freshly isolated mice cardiomyocytes, we can observe that AngII-induced cytoplasmic TR3 expression (Fig 4A, bottom), which is similar to the results from H9C2 cells and NRCMs (Fig. 4A, top & middle).

Answer to the Reviewer 3

1 ***mTOR forms two complexes: mTORC1 (rapamycin-sensitive) and mTORC2 (rapamycin-insensitive). In the manuscript, they did not examine or discuss anything about mTORC2 signaling. Akt, a downstream molecule of mTORC2, plays an important role in cardiac hypertrophy. In addition, previous reports demonstrated that long-term rapamycin treatment inhibits Akt activation. They should evaluate mTORC2 as well as mTORC1 activation.***

According to your suggestion, we have described the mTORC2 signaling in “Introduction”. (page 3-4) In addition, phospho-Ser473 of AKT was used to monitor the activity of mTORC2. As shown in Fig, S6B, after AngII treatment, the levels of AKT phosphorylation showed no difference in MEFs and $TR3^{-/-}$ MEFs or NRCMs and TR3-KD NRCMs, even in the presence of rapamycin, indicating that TR3 does not participate in Ang-II-induced mTORC2 activation. However, in the same condition, mTORC1 (monitored by S6K1 phosphorylation) was activated by AngII, and rapamycin showed an obvious inhibition on AngII-induced mTORC1 activation.

2 ***In Figure 1, while they demonstrated expression level of TR3 in Ang-II-treated WT mice, there are no data to compare the protein levels of TR3 between WT and KO mice. This is also applied to the experiments with rats infected with lentivirus carrying siTR3 (Figure 3).***

Protein levels of TR3 in WT and TR3-KO mice (Fig. S1A), NRCMs and TR3-KD NRCMs (Fig. S3F), MEFs and $TR3^{-/-}$ MEFs (Fig. 3D), or the left ventricle of WT and that of TR3-KD rats (Fig. S2A) are indicated.

3. ***Although the authors pointed about tissue specificity for studying the signaling pathway in the heart, they did not confirm the cardiac-specific effect of siTR3 (they stated "organ-specific" in the text). They should show protein level of TR3 in the heart and other tissues to demonstrate the organ specificity in the system with lentiviruses.***

In order to demonstrate the organ-specific knockdown of TR3, we dissected the left ventricle and other tissues from mice to detect the TR3 expression levels. Figure S2A showed that TR3 was efficiently knocked down in the left ventricle of rats, but not in other tissues including right ventricle, liver, muscle and kidney.

4 ***In Figure 2D, since the results of Lung/Body weight look marginal, those do not indicate the beneficial effect of TR3 gene silencing on cardiac function in Ang II-induced cardiac hypertrophy. If they want to discuss about cardiac function, they should perform physiological assays such as echocardiography.***

As you mentioned, the changes in lung weight/body weight before and after AngII treatment is indeed very faint, so we deleted this result. Instead, lots of echocardiographic parameters were further detected and summarized in Tab. 1.

5 ***In Figure 1D, quantitative assay of cross section of cardiomyocytes should be performed with the tissue samples.***

To quantify the cross section of cardiomyocytes, we calculate the mean cross-sectional area of cardiomyocytes by using ImageJ software. This method has been reported by several groups [Duan, S., et al. *Circ Res*, 2005, 97(4): 372-9; Tirziu, D., et al. *J Clin Invest*, 2007, 117(11): 3188-97; Kim, Y., et al. *J Clin Invest*, 2008, 118(1): 124-32]. Fig. 1D showed that the mean cross sectional area (mm^2) of WT cardiomyocyte is significantly larger than that of TR3-KO cardiomyocytes after AngII administration.

6 ***In Figure 1C, 2A, 2B, and 2D, a comparison between WT and KO should be important.***

Comparisons between WT and KO after AngII administration were conducted using ANOVA statistical method and there is a significant difference between them (Fig. 1C, 2A, 2B). The ANOVA statistical method is mentioned in Meterials and Methods (page 24).

7 ***In Figure 2C, the authors should describe how many nuclei were used for the quantitative assay of TUNEL.***

300 nuclei in each section are counted for the quantification of TUNELL assay, which is described in page 31.

8. ***In Figure 6, to demonstrate the effect of rapamycin, Western blotting showing phosphorylation levels of S6K1 and Akt should be performed. This is applied to Supplementary Fig 3D too.***

To reveal the effect of rapamycin on the activities of mTORC1 and mTORC2, we determined the levels of phospho-Thr389 of S6K1 (indicator for mTORC1 activity) and that of phospho-Ser473 of AKT (indicator for mTORC2 activity) in MEFs and NRCMs and their corresponding TR3-KO and TR3-KD cells. The results showed that rapamycin remarkably decreased the phospho-Thr389 of S6K1 but not phospho-Ser473 of AKT in both MEFs and NRCMs (Fig. S6B). Similar results were also obtained from the sample of mice (Fig. S3E). The results further demonstrate that mTORC1 is rapamycin-sensitive and mTORC2 is rapamycin-insensitive.

*9. **In Figure 6, representative photos of NRCM and MEF should be helpful to demonstrate the effect of TR3 in Agn II-induced hypertrophy. For demonstrating cardiomyocyte hypertrophy in NRCM, immunohistochemistry with sarcomeric proteins such as alpha-actinin is broadly used.***

According to the suggestion, immunofluorescence assays using anti-actinin (for NRCMs) and FITC-conjugated phalloidin (for MEFs) were carried out. As shown in Fig. S6A, cell size was apparently enlarged after AngII treatment in NRCMs and $TR3^{+/+}$ MEFs. However, in TR3-KD NRCMs and $TR3^{-/-}$ MEFs, cell size was comparable before and after AngII treatment. Of noted, when these cells were co-treated with rapamycin, cell size was apparently decreased even in the presence of AngII, which is consistent with the reports [Malhowski, A.J., et al. *Hum Mol Genet*, 2011, 20(7): 1290-305].

10. ***In Supplementary Fig 1B, it is not clear to observe localization and expression level of TR3. The authors should try immunohistochemical staining with different fluorescent colors for anti-tropomycyin and ant-TR3.***

According to the suggestion, we carry out immunofluorescent staining using anti-TR3 and anti-tropomycin antibodies in the same section. The nuclei were stained by DAPI simultaneously. The results not only revealed that TR3 colocalized with tropomyosin-positive cardiomyocytes, but also demonstrated that the expression of cytoplasmic TR3 was obviously elevated in tropomycin positive cells after AngII induction, suggesting AngII-induced TR3 expression indeed occurred in cardiomyocytes (Fig. S1B).

11. *On page 15 (Figure 6C), they investigated the effect of TR2 in apoptosis. ROS is known to induce apoptosis (but not an index). If the authors want to investigate apoptotic change in the setting. They should study caspase signaling pathways. As mentioned above, Akt is a key molecule of the mTOR signaling pathway: the upstream molecule of mTORC1 signaling and the downstream molecule of mTORC2. It is well known that Akt inhibits apoptosis in cardiomyocytes. Thus they need to examine Akt activation as well if they discuss the effect of TR3 in Ang II-induced apoptosis.*

ROS is a cause of apoptosis but also an indirect indicator of mTORC1 activity [Lee, J.H., et al. *Science*, 2010, 327(5970): 1223-8]. The purpose in this study is to verify the TR3 effect on mTORC1 activity through detection of ROS level, rather than the function of TR3 on AngII-induced apoptosis. Therefore, we have rewritten our sentence as "ROS level is additionally measured as an indicator of mTORC1 activity " (page 17).

Since Akt is unlikely to involve in TR3-mediated cardiac hypertrophy in our study, we do not examine AKT effect on apoptosis.

2nd Editorial Decision 10 September 2012

Thank you for the submission of your revised manuscript "The orphan receptor TR3 participates in angiotensin II-induced cardiac hypertrophy by controlling mTOR signaling" to EMBO Molecular Medicine. We have now received the enclosed reports from the referees whom we asked to re-assess it.

As you will see, the Reviewers acknowledge that the manuscript was significantly improved during revision. However, they still raise concerns that should be convincingly addressed. Since we do acknowledge the potential interest of your findings, we would be willing to consider a revised manuscript with the understanding that the referee concerns must be convincingly and conclusively addressed.

Specifically, both Reviewers highlight that the statistical analysis has to be improved in addition to some technical concerns.

Revised manuscripts should be submitted latest within three months of a request for revision; they will otherwise be treated as new submissions, unless arranged otherwise with the editor.

I look forward to seeing a revised form of your manuscript as soon as possible.

Yours sincerely,

Editor
EMBO Molecular Medicine

***** Reviewer's comments *****

Referee #1 (Comments on Novelty/Model System):

This manuscript presents what can be understood as a novel regulatory mechanism of mTOR pathway by AII via TR3 orphan nuclear receptor. Experiments were performed to show that cardiomyocyte TR3 mediates the pathological hypertrophy induced AII. Furthermore, data was provided indicating that TR3 upregulares the mTORC1 pathway by reducing the levels of TSC2. By performing additional experiments suggested by the reviewers the authors have made some progress in improving the overall quality of the data and consequently of the manuscript. However this reviewer feels that clarifications are still needed before the manuscript can be accepted for publication, which I list below in my comments to the authors.

Referee #1:

The new version of the manuscript by Wang et al is substantially improved as compared to the first version. However, this reviewer feels that are still points that need clarification, as follows:

1. The text needs further revision. To mention a few problems:
Page 5; Line 20- the sentence starting as "It is possible that TR3 directly bound..." needs to be rephrased.
Page 6; 2nd paragraph - I suspect that the correct sentence is "...in the transverse aorta constriction (TAC) mouse model, which induced..."
Page 6; 2nd paragraph - Revise sentence which starts as "...We first established a model...". Plus, I change to "osmotic minipump".

2. There are still problems with the statistical comparisons. Comparisons against the controls should be included in the data shown in table 1, Fig. 1B and Fig. 2D.

3. Figure 1C - Data representing the dry weight versus wet weight of the hearts should not be so discrepant. The possible error introduced by the volume of water should be similar for all groups anyway. Please, clarify whether the new "dry weight" data were obtained from a new group of mice and if it is true, please add the wet weight for comparisons.

4. Figure 4. TR3 seems to migrate differently in the gels from H9C2, NRCM and adult cardiomyocytes. Plus, one would expect the amount of TR3 in the total extract greater than that on the individual fractions. It surprises me that there was no cytosolic contamination (tubulin) in the nuclear fraction. The authors need to provide pictures of the entire gels of the blots as supplementary figures.

Referee #3:

The manuscript is substantially improved. The authors performed additional experiments according to the author's suggestions, including Akt phosporylation, TR3 expression, quantitative assay in area of myocyte cross-section, protein levels of TR3 expression in multiple tissues in the organ-specific

knockdown of TR3 in rats and immunohistochemistry with different secondary antibodies. Introduction, Results and Methods in manuscript were significantly improved as well. The revised manuscript strengthens evidences in the role of TR3-mediated mTOR signaling pathway in AngII-induced cardiac hypertrophy.

Minor suggestion:
- In Table 1, statistical analysis of the %FS between sham and AngII-treated-TR3-WT mice should be confirmed. It looks statistically different.

1st Revision - authors' response 08 October 2012

Answer to the Referee #1:

1. The text needs further revision. To mention a few problems:
Page 5; Line 20- the sentence starting as "It is possible that TR3 directly bound..." needs to be rephrased.
Page 6; 2nd paragraph - I suspect that the correct sentence is "...in the transverse aorta constriction (TAC) mouse model, which induced..."
Page 6; 2nd paragraph - Revise sentence which starts as "...We first established a model...". Plus, I change to "osmotic minipump".

We have revised these sentences as you mentioned above. In addition, we sent the manuscript for further revision by Nature Publishing Group Language Editing (NPGLE). We also enclosed the certification provided by NPGLE.

2. There are still problems with the statistical comparisons. Comparisons against the controls should be included in the data shown in table 1, Fig. 1B and Fig. 2D.

According to your suggestion, we have made the comparisons of AngII-treated groups against their respective controls in wild-type mice in the Table 1, Fig. 1B and Fig. 2D.

3. Figure 1C - Data representing the dry weight versus wet weight of the hearts should not be so discrepant. The possible error introduced by the volume of water should be similar for all groups anyway. Please, clarify whether the new "dry weight" data were obtained from a new group of mice and if it is true, please add the wet weight for comparisons.

Initially, we measured heart wet weight by artificially squeezing watery blood out from the chamber of heart, this process may lead to an error. In the revised version, this artificial step was canceled; instead, we dried heart at 60°C for 48 hours as described in literatures (*Circulation*. 1995; 91: 161-170). We think these "dry weight" data are more creditable than the original "wet weight" data.

The "dry weight" data is obtained from a new group of mice. However, we neglected measuring the wet weight. We feel very sorry that your requested data is not available. Hope you can understand. Thanks.

4. Figure 4. TR3 seems to migrate differently in the gels from H9C2, NRCM and adult cardiomyocytes. Plus, one would expect the amount of TR3 in the total extract greater than that on the individual fractions. It surprises me that there was no cytosolic contamination (tubulin) in the nuclear fraction. The authors need to provide pictures of the entire gels of the blots as supplementary figures.

The fact that TR3 migrates differently in the gels from H9C2, NRCM and adult cardiomyocytes may be due to the different samples from different tissues. Moreover, the usage of different TR3 antibodies for different tissues/cell lines may be another reason.

The result that the amount of TR3 in the total extract seems no greater than that on the individual fractions may be caused by the too long exposure time, which could impair the difference in each lane. So, we repeated these experiments, and quantified the amounts of TR3 in each lane by ImageJ software (indicated at the bottom).

Using our fractionation methods, it is easy to get pure cytosolic and nuclear fractions. There is no cytosolic contamination (tubulin) in the nuclear fraction. Please refer our protocol at the section of supplementary methods. Please see the entire gels of original blots in supplementary Fig. 7.

Answer to the Referee #3:

In Table 1, statistical analysis of the %FS between sham and AngII-treated-TR3-WT mice should be confirmed. It looks statistically different.

Yes, it is statistically significant. As an indicator of cardiac function, the % FS changes remarkably in WT mice after AngII administration. Here we are sorry that the statistical analysis on % FS was missed in the previous submitted manuscript. Thank you very much for your remind.
