# Supplementary material for: The orphan receptor TR3 participates in angiotensin II-induced cardiac hypertrophy by controlling mTOR signalling
Source: EMBO Mol Med. 2012 Nov 29;5(1):137–48. doi: 10.1002/emmm.201201369 (PMC3569659; doi:10.1002/emmm.201201369)

Supplementary Information

The orphan receptor TR3 participates in angiotensin II-induced cardiac hypertrophy by controlling mTOR signaling

Rong-Hao Wang[1]*, Jian-Ping He[1]*, Mao-Long Su[2]*, Jie Luo[1]*, Ming Xu[3], Xiao-Dan Du[1], Hang-Zi Chen[1], Wei-Jia Wang[1], Yuan Wang[1], Nan Zhang[2], Bi-Xing Zhao[2], Wen-Xiu Zhao[2], Zhong-Gui Shan[2], Jia-Huai Han[1], Chawnshang Chang[4], Qiao Wu[1]**

Table of contents

Supplementary Methods

Supplementary Figure Legends

Supplementary References

Supplementary Figure S1

Supplementary Figure S2

Supplementary Figure S3

Supplementary Figure S4

Supplementary Figure S5

Supplementary Figure S6

Supplementary Methods

Co-immunoprecipitation assay (co-IP)

Cells or heart tissues were lysed in an ice-cold lysis buffer (20 mM Tris-HCl pH 7.5, 150 mM NaCl, 1 mM EDTA, 1 mM EGTA, 1% Triton X-100, 2.5 mM sodium pyrophosphate, 1 mM β-glycerophosphate, 1 mM Na_3VO_4 and 1 mM phenylmethylsulfonyl fluoride). After centrifugation, the supernatant was incubated with the corresponding antibodies and protein A/G Sepharose beads (Santa Cruz) for 3 h and subsequently centrifuged for 3 min at 1000 *g*. The immunoprecipitates were collected and washed three times with the lysis buffer and then subjected to a western blotting analysis.

Western blot analysis

Cells or tissues were lysed in an ice-cold lysis buffer as described above. After centrifugation, equal amounts of the protein were loaded for electrophoresis on 6%-15% denaturing SDS-PAGE gels. The blots were probed with the primary antibodies overnight at 4 °C, followed by incubation with the appropriate secondary antibodies at room temperature for 1 h. The following primary antibodies were used: anti-p70 S6 Kinase (Cell Signaling), anti-phospho-p70 S6 kinase (Thr389) (Cell Signaling), anti-TSC1 (Santa Cruz), anti-TSC2 (Santa Cruz), anti-Nur77 (Cell Signaling) for human TR3, anti-Nur77 (Epitomics) for mouse TR3, anti-Nur77 (Abcam or Epitomics) for rat TR3, anti-tubulin (Sigma), anti-HA (Sigma), anti-Flag (Sigma), anti-Myc (Roche), anti-histone4 (Abcam), anti-actinin (Sigma), anti-tropomyosin (Sigma), and anti-phospho-AKT (Ser473) (Cell signaling). The antibody

reactivity was detected with an enhanced chemiluminescence kit according to the manufacturer's instructions.

Ubiquitination assay

Myc-ubiquitin was transfected into the cells using a Turbofect transfection agent (Thermo Fisher Scientific). After treatment with MG132 (10 μM, 3 h), endogenous TSC2 was precipitated in a RIPA lysis buffer (50 mM Tris, 150 mM NaCl, 1% Triton X-100, 0.1% SDS, 1% sodium deoxycholate, 1 mmol/L β-glycerophosphate, 1 mmol/L Na_3VO_4 and 1 mM phenylmethylsulfonyl fluoride) and then subjected to western blotting.

Nuclear and cytosol fractionation

To fractionate cytosol fraction of heart tissue, tissues were homogenized in isolation buffer (70 mmol/L sucrose, 190 mmol/L D-mannitol, 20 mmol/L Hepes, 0.2 mmol/L EDTA) using a Dounce homogenizer. Nuclear fractions were separated by centrifugation at 600 g for 10 min. After complete clearance of nuclear fractions with isolating buffer, cytosolic fractions were obtained by centrifugation at 100,000 g for 60 min.

To fractionate nuclear and cytosol fraction of cells, cells were trypsinized and washed once with PBS. An aliquot of cells (1/10) was lysed with 100 μL ice-cold lysis buffer (20 mM Tris-HCl pH 7.5, 150 mM NaCl, 1 mM EDTA, 1 mM EGTA, 1% Triton X-100, 1 mM phenylmethylsulfonyl fluoride) as total input, and the remaining (9/10) was suspended in 200 μL ice-cold Buffer A (10 mM HEPES, pH7.9, 10 mM KCl, 0.1 mM EDTA, 0.1 mM EGTA, 0.15% NP-40, 1 mM phenylmethylsulfonyl fluoride) on ice for 15 min. After centrifuged at

12000 g for 1 min at 4 ℃, the supernatants (cytosol fractions) were collected. The pellets (nuclear fractions) were resuspended and incubated with 800 μL buffer A for 5 min on ice and centrifuged at 12000 g for 1 min at 4 ℃. This wash procedure was repeated for 4 times. After centrifugation, the pellets were lysed with 100μL Buffer B (20 mM HEPES, pH7.9, 0.4 mM NaCl, 1 mM EDTA, 1 mM EGTA, 0.5% NP-40, 1 mM phenylmethylsulfonyl fluoride). The protein concentration was measured and equal amounts of proteins were subjected to western blotting analysis.

Isolation of adult mice cardiomyocytes

The isolation of adult mice cardiomyocytes was completed in a Langendorff perfusion system (Tada et al, 2000). Briefly, mice were intraperitoneally injected with 0.2 ml heparin (400 U/ml) and anesthetized. The heart was excised quickly and placed in an ice-cold Ca^{2+} free tyrode buffer (135 mM NaCl, 4 mM KCl, 1 mM $MgCl_2$, 10 mM HEPES, 0.33 mM NaH_2PO_4, 10 mM glucose, 10 mM 2,3-butanedione monoxime (Sigma) and 5 mM taurine (Sigma)). The cannulation of the heart was completed within 90 s, and the heart was perfused with Ca^{2+} free tyrode buffer for 3 min at the rate of 3 ml/min and then digested by a digestion solution (0.5 mg/ml collagenase B (Roche), 0.3 mg/ml collagenase D (Roche) and 0.02 mg/ml protease XIV (Sigma) dissolved in the tyrode buffer) for 10 min. After digestion, the ventricles were cut and pipetted several times, and then the cardiomyocytes were transferred to pre-coated laminin dishes (Sigma) for further use.

Isolation of neonatal rat cardiomyocytes

Neonatal rat cardiomyocytes were isolated according to a previously described method (Chandrasekar et al, 2005) with certain modifications. Briefly, the hearts were collected from 3-day-old neonatal rats and cut into small pieces. The pieces were washed twice with an ADS buffer (11.6 mM NaCl, 1.8 mM HEPES, 0.1 mM NaH_2PO_4, 0.5 mM KCl, 83 μM $MgSO_4$, and 55 mM glucose) and subjected to a series of digestions with 80.6 units/ml type 2 collagenase (Gibco) and 62.5 μg/ml trypsin (Bio Basic Inc.) in the ADS buffer. The pieces of tissue were incubated with the enzyme solution and agitated gently at 37°C for 20 min for each digestion. After eight repetitions, all of the supernatants, except for those collected from the first round of digestion, were collected in newborn calf serum and centrifuged at 250 g for 10 min. The resulting pellets were resuspended in a culture medium (4 parts DMEM, 1 part M199, 10% horse serum, 5% fetal bovine serum, 100 U/mL penicillin and 100 U/mL streptomycin), and the suspensions were passed through a 100-μM cell strainer (BD Bioscience) into a new cell-culture dish to remove the non-cardiomyocytic cells by differential attachment at 37°C for 90 min. The nonattached cells (cardiomyocytes) were centrifuged at 250 *g* for 10 min, resuspended in culture medium, counted with a hemocytometer and seeded at the required density on 1% gelatin-coated dishes. The cells were used for western blotting detection or immunofluorescent staining.

Immunofluorescent staining

The cultured cells were seeded on coverslips. After fixation in 4% paraformaldehyde, the cells were blocked in PBS containing 10% BSA at room temperature for 30 min. The cells were then incubated with antibodies as required for 3 h, followed by treatment with

fluorescent isothiocyanate-conjugated (FITC) (Pharmingen) or Texas Red-conjugated (Pharmingen) secondary antibodies for an additional 3 h. The nuclei were stained with 4',6-diamidino-2-phenylindole (DAPI). Images were obtained using a confocal microscope (Leica, TCS SPISE).

Flow cytometry

(1) Cell size and protein/DNA ratio measurement

Cells were trypsinized, washed with PBS, and fixed in ice-cold 70% PBS-buffered ethanol at 4 °C overnight. The fixed cells were centrifuged at 800 *g* for 10 min and washed twice with 1 mL of PBS.

For the cell size measurement, the cells were resuspended in 500 μL of PBS containing 100 U/mL RNaseA and 50 μg/mL PI at 37 °C for 30 min of dark incubation. The cells were then analyzed on a Coulter Epics XL (Beckman) that was gated to measure the forward scatter (FS) of the G0/G1 cells. The results were further processed with WinMDI 2.8 software.

For the protein/DNA calculation, the cells were resuspended in 500 μL of PBS containing 100 U/mL RNaseA and 1 μg/mL FITC at 37 °C for a 2 h dark incubation and stained with PI (50 μg/mL) at the same temperature for another 30 min. The prepared cells were subjected to flow cytometry. The mean fluorescence from 20,000 cells was measured for FITC (protein) at 525 nm and PI (DNA) at 620 nm.

(2) Intracellular ROS measurement

Cells were plated on 35 mm dishes at 80% confluence, washed with PBS and cultured in 500 nM AngII-containing DMEM for 3 h. To permit probe loading, DCFH-DA (Sigma)

was added to a final concentration of 10 μmol/L 30 min before the end of the treatment. The cells were then trypsinized, washed twice with PBS, resuspended in 500 μL PBS and subjected to flow cytometry analysis. The relative fluorescence from 30,000 cells was recorded at 530 nm, and the results were analyzed using WinMDI 2.8 software.

Real-time PCR

The total RNA was isolated using RNAsimple (Tiangen) and reverse-transcribed by RevertAid reverse transcriptase (Fermentas). Real-time PCR was performed using FastStart SYBR Green mix (Roche) according to the manufacturer's instructions. The following primers were used for amplification:

For *ANP*: forward 5’-TCGTCTTGGCCTTTTGGCT-3’;
reverse 5’-TCCAGGTGGTCTAGCAGGTTCT-3’;
For *BNP*: forward 5’ -AAGTCCTAGCCAGTCTCCAGA-3’;
reverse 5’- GAGCTGTCTCTGGGCCATTTC-3’;
For *MHC*: forward 5’-TGCAAAGGCTCCAGGTCTGAGGGC-3’;
reverse 5’-GCCAACACCACCCTGTCCAAGTTC-3’;
For rat *TR3*: forward 5’-TGTTGATGTTCCTGCCTTTGC-3’;
reverse 5’-TGCGGTTCTGCAGCTCCT-3’;
For *GAPDH*: forward 5'-GACCACAGTCCATGCCATCAC-3';
reverse 5'-CATACCAGGAAATGAGCTTGAC-3'.

Immunohistochemistry

Freshly dissected hearts were fixed in 4% paraformaldehyde, embedded in paraffin and

sectioned at a thickness of 5 μm. After dewaxing and rehydration, the sections were stained with hematoxylin and eosin to examine morphological changes.

For immunohistochemical and immunofluorescent staining in the heart tissue, the deparaffinized and rehydrated sections were pretreated with a peroxidase blocking buffer (Maxim) for 20 min at room temperature. Antigen retrieval was performed by boiling the sections in a citrate buffer (pH 6.0) for 20 min. After a preincubation with the blocking buffer (5% normal goat serum in PBS) for 1 hour at RT, incubation with an antibody was performed at room temperature for 2 h. The Ultrasensitive S-P kit (Maxim) was used as a secondary reagent for the immunohistochemical analysis, and a FITC-conjugated or Texas Red-conjugated secondary antibody was used for immunofluorescent staining.

To assess myocardial fibrosis, a Masson trichrome stain kit (Maxim) was used according to the manufacturer's recommendation. The cytosol and fibrotic areas were stained red and blue, respectively.

A TUNEL kit (Keygen) was used to examine apoptotic cells. Briefly, deparaffinized and rehydrated sections were digested with 20 μg/mL proteinase K at 37°C for 30 min. The endogenous peroxidase was blocked by 3% H_2O_2 in methanol at room temperature for 10 min. The samples were incubated with TdT and biotin-conjugated dUTP in an equilibration buffer at 37°C for 1 h, followed by incubation with streptavidin-conjugated horseradish peroxidase (HRP). The apoptotic nuclei were visualized using the 3,3'-diaminobenzidine (DAB) reaction and counterstained with hematoxylin (Sigma). The TUNEL-positive and TUNEL-negative nuclei were counted; the results are given as the percentage of TUNEL-positive nuclei.

Electrophoresis mobility shift assay (EMSA)

An EMSA was performed as previously described (Liu et al, 2010) using a biotin-labeled double-stranded TR3 binding element (5’-GATCCGTGACCTTTATTCTCAAAGGTCA-3’). For the up-shift experiment, 8 μg of the nuclear extract was pre-incubated with 200 ng of an anti-TR3 antibody (Cell Signaling) for 1 h before incubation with the probe. Competition was performed using a 100-fold excess of non-labelled probes.

Measurement of the mean cardiomyocyte size and myocardial fibrotic area

ImageJ software (version 1.46p, http://rsb.info.nih.gov/ij/) (NIH) was applied for the measurement as previously described (Kim et al, 2008). For cross-sectional cardiomyocyte area measurement, H&E stained sections were used. The cross-sectional areas of approximately 100 cardiomyocytes were analyzed for each group (n=3). For isolated adult cardiomyocyte size measurement, isolated cells were stained with actinin and about 10 chosen fields for each group (n=3) were measured. For the measurement of the fibrotic area, Masson trichrome-stained sections were used, and the fibrotic proportion was calculated by dividing the blue area (collagen) by the whole selected area. Approximately 10 chosen fields were analyzed for each group (n=3).

Supplementary Figure Legends

Supplementary Figure S1

(A) TR3 is not involved in AngII-induced hypertension. Top, The endogenous TR3 expression in WT and TR3-KO mice was detected by western blotting with an anti-TR3 antibody. Bottom, The blood pressure was measured using a Softron Indirect Blood Pressure Meter. WT and TR3-KO mice (n=15) were continuously administered AngII for 4 weeks via a micro-osmotic pump, and the blood pressure was measured weekly.

(B) TR3 expression in cardiomyocytes detected by immunohistochemical staining. The sections were from the hearts of the WT mice as described above. The mice were treated with AngII for 4 weeks. Tropomyosin was used to label the cardiomyocytes. Top, DAB was used as a substrate to reveal the stained proteins. Bottom, FITC-conjugated and Texas Red-conjugated secondary antibodies were applied to indicate TR3 (green) and tropomyosin (red), respectively. The nuclei were stained with DAPI (blue). The images were obtained using confocal microscopy. The arrows show the overlap of TR3 with the nucleus. After the administration of AngII, the expression of cytoplasmic TR3 was increased, as indicated by a yellow color compared with the sham-operated heart section in the immunofluorescent analysis (scale bar=50 μm).

(C) The effect of losartan or propranolol on AngII-induced blood pressure. During the AngII infusion, the WT mice (n=5) received losartan or propranolol via the drinking water for 2 weeks, and the blood pressure was measured. The same volume of 0.9% saline plus 0.01 M acetic acid was substituted for AngII in the sham-operated WT mice. **: p<0.01, NS:

non-significant.

Supplementary Figure S2

(A) The TR3 expression in the different tissues. Endogenous TR3 was specifically knocked down in the left ventricles of the rats using lentivirus-based RNA interference, and the control rats were injected with viruses expressing scrambled siRNA. Proteins from the different tissues as indicated were prepared and subjected to TR3 detection.

(B) The blood pressure of the WT and TR3-KD rats, in which TR3 was knocked down in the left ventricles, is shown before and after AngII administration.

Supplementary Figure S3

(A) The detection of TR3 transcriptional activity before and after AngII treatment. H9C2 cells were transfected with the reporter gene NurRE (a TR3 response element). After the transfection, the cells were treated with AngII for different intervals and collected for a luciferase assay. The transfection of HA-TR3 was used as a positive control.

(B) An EMSA analysis is performed to exclude the effect of AngII on TR3 DNA binding. After 3 h of AngII treatment in H9C2 cells, the nuclear extracts were prepared and analyzed by EMSA using a biotin-labeled TR3 response element as a probe. The specific binding of TR3 to DNA was confirmed by incubation with a non-labeled competitor (no band) and an anti-TR3 antibody (up-shifted band).

(C) The mRNA level of *TR3* detected by real-time PCR in H9C2 cells and NRCMs before and after AngII administration for the indicated intervals.

(D) The effect of AngII on the half-life of the TR3 protein in H9C2 cells. The cells were transfected with TR3 and then treated with CHX (100 μg/ml) alone or in combination with 500 nM AngII for the indicted amounts of time. The protein levels of TR3 were determined using western blotting (top) and quantified using densitometry (bottom). Three independent trials were performed for each experiment. The bar represents the mean ± SD of the three trials.

(E) Masson’s trichrome staining to demonstrate the effect of rapamycin on the AngII-induced cardiac fibrosis (left). A Masson’s trichrome staining kit was used to detect and display the deposited collagen in the heart sections (scale bar=50 μm). The effect of rapamycin on mTORC1 activity was shown (right). The proteins were prepared from the left ventricle of the mice and then subjected to western blotting to detect the phosphorylation of S6K1; tubulin was used as loading control.

(F) The expression of TR3 in NRCMs and TR3-KD NRCMs. The endogenous TR3 was specifically knocked down using lentivirus-based RNA interference, and the control cells were transfected with scrambled-siRNA. The TR3 level was determined by western blotting.

(G) TR3 was detected upstream of the mTORC1 pathway. The H9C2 cells were pretreated with rapamycin for 1 h and then treated with 500 nm AngII for 3 h. The cells were collected and subjected to western blotting analysis for the detection of TR3.

Supplementary Figure S4

(A) The localization of TR3 in the nucleus and cytoplasm of human samples from the left ventricle septum. Representative left ventricular samples from normal and hypertrophic

human hearts were subjected to immunohistochemical analysis using a TR3 antibody (scale bar=50 μm).

(B) TR3 interacts with TSC1 or TSC2 in 293T cells. After the co-introduction of HA-TSC2 or Myc-TSC1 with Flag-TR3 into 293T cells, the cells were lysed and were subjected to co-IP assays. Reciprocal precipitations were performed.

(C) TR3 colocalized with TSC1 or TSC2 as shown by immunofluorescent staining. H9C2 cells were fixed and then immunostained with TR3 and TSC1 or TSC2 antibodies, followed by staining with FITC-conjugated and Texas Red-conjugated secondary antibodies. Images were obtained using confocal microscopy.

(D) Two-step immunoprecipitation is used to detect the TR3-TSC1-TSC2 complexes. The procedure for the two-step immunoprecipitation is shown on the left. Flag-TR3 and HA-TSC2 were transfected into the 293T cells with or without Myc-TSC1 (control). The first immunoprecipitation assay was performed using an anti-Myc antibody, and the precipitate was eluted with a Myc peptide. A second round of immunoprecipitation using anti-Flag antibody. The components obtained from each step of the immunoprecipitation were identified by immunoblotting with anti-Myc, anti-Flag and anti-HA to detect TSC1, TR3 and TSC2, respectively.

(E) The ligand-binding domain of TR3 is the essential domain for the interaction with TSC. The full-length TR3 or its deletion mutants (shown in schematic diagrams, top) were transfected together with HA-TSC2 or Myc-TSC1. After the transfection, the cell lysates were subjected to immunoprecipitation. TR3 and its deletion mutants were detected using an anti-Flag antibody.

Supplementary Figure S5

(A) TR3 degrades TSC2 but not TSC1 in a concentration-dependent manner. HA-TSC2 and Myc-TSC1 were transfected into the 293T cells along with different concentrations of Myc-TR3 or HA-TR3. After the transfection, the cell lysates were prepared, and HA-TSC2 and Myc-TSC1 were detected using HA and Myc antibodies, respectively. Tubulin was used as a loading control.

(B) TR3 stabilizes the TSC2 protein level. CHX (100 μg/ml) was used to treat $TR3^{+/+}$ and $TR3^{-/-}$ MEFs for the indicated durations. The cells were collected and subjected to TSC2 detection. The experiment was repeated for three independent trials. The bar represents the mean ± SD of the three trials.

(C) The mapping of the TSC2 region required for TR3 binding. Various deletion mutants of TSC2 (shown in the schematic diagrams, top) were transfected with Flag-TR3 into the 293T cells. After transfection, the cell lysates were prepared for immunoprecipitation using a Flag antibody, and the immunoprecipitates were subjected to western blotting using an HA antibody.

Supplementary Figure S6

(A) Representative images of $TR3^{+/+}$ and $TR3^{-/-}$ MEFs, NRCMs and TR-KD NRCMs. The cells were treated with 10 nM rapamycin and 500 nM AngII for 48 h. Anti-α-actinin (red color) was used to mark the NRCMs, and FITC-conjugated phalloidin (green color) was applied to indicate the MEFs.

(B) TR3 does not participate in AngII-induced mTORC2 activity. The $TR3^{+/+}$ and $TR3^{-/-}$ MEFs (left) or the NRCMs and TR-KD NRCMs (right) were cotreated with 10 nM rapamycin and 500 nM AngII for 48 h and then collected for the detection of the AKT phosphorylation (Ser-473). S6K1 phosphorylation (Thr-389) was used as a positive control, and tubulin was used as a loading control.

References

Chandrasekar B, Mummidi S, Claycomb WC, Mestril R, Nemer M (2005) Interleukin-18 is a pro-hypertrophic cytokine that acts through a phosphatidylinositol 3-kinase-phosphoinositide-dependent kinase-1-Akt-GATA4 signaling pathway in cardiomyocytes. *J Biol Chem* **280:** 4553-4567

Kim Y, Phan D, van Rooij E, Wang DZ, McAnally J, Qi X, Richardson JA, Hill JA, Bassel-Duby R, Olson EN (2008) The MEF2D transcription factor mediates stress-dependent cardiac remodeling in mice. *J Clin Invest* **118:** 124-132

Liu JJ, Zeng HN, Zhang LR, Zhan YY, Chen Y, Wang Y, Wang J, Xiang SH, Liu WJ, Wang WJ, Chen HZ, Shen YM, Su WJ, Huang PQ, Zhang HK, Wu Q (2010) A unique pharmacophore for activation of the nuclear orphan receptor Nur77 in vivo and in vitro. *Cancer Res* **70:** 3628-3637

Tada H, Thompson CI, Recchia FA, Loke KE, Ochoa M, Smith CJ, Shesely EG, Kaley G, Hintze TH (2000) Myocardial glucose uptake is regulated by nitric oxide via endothelial nitric oxide synthase in Langendorff mouse heart. *Circ Res* **86:** 270-274

Supplementary Fig. S1

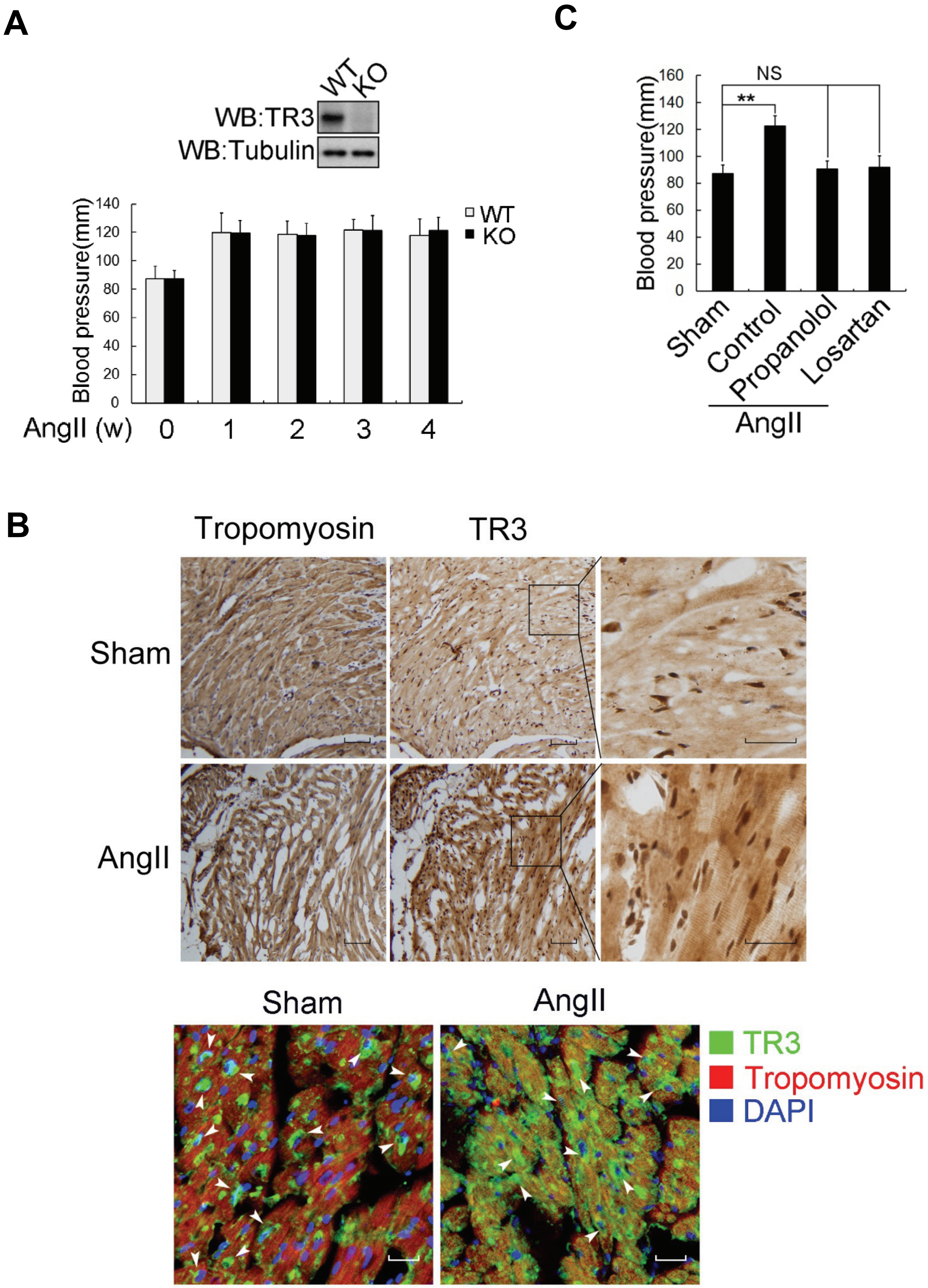

Supplementary Fig. S2

A

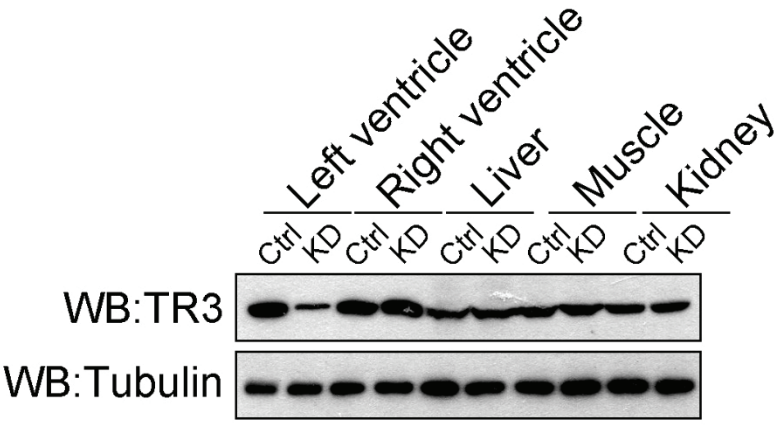

B

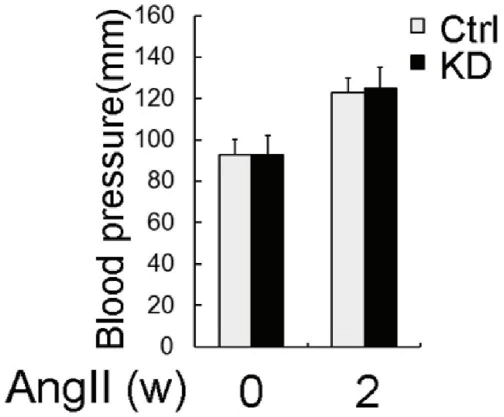

Supplementary Fig. S3

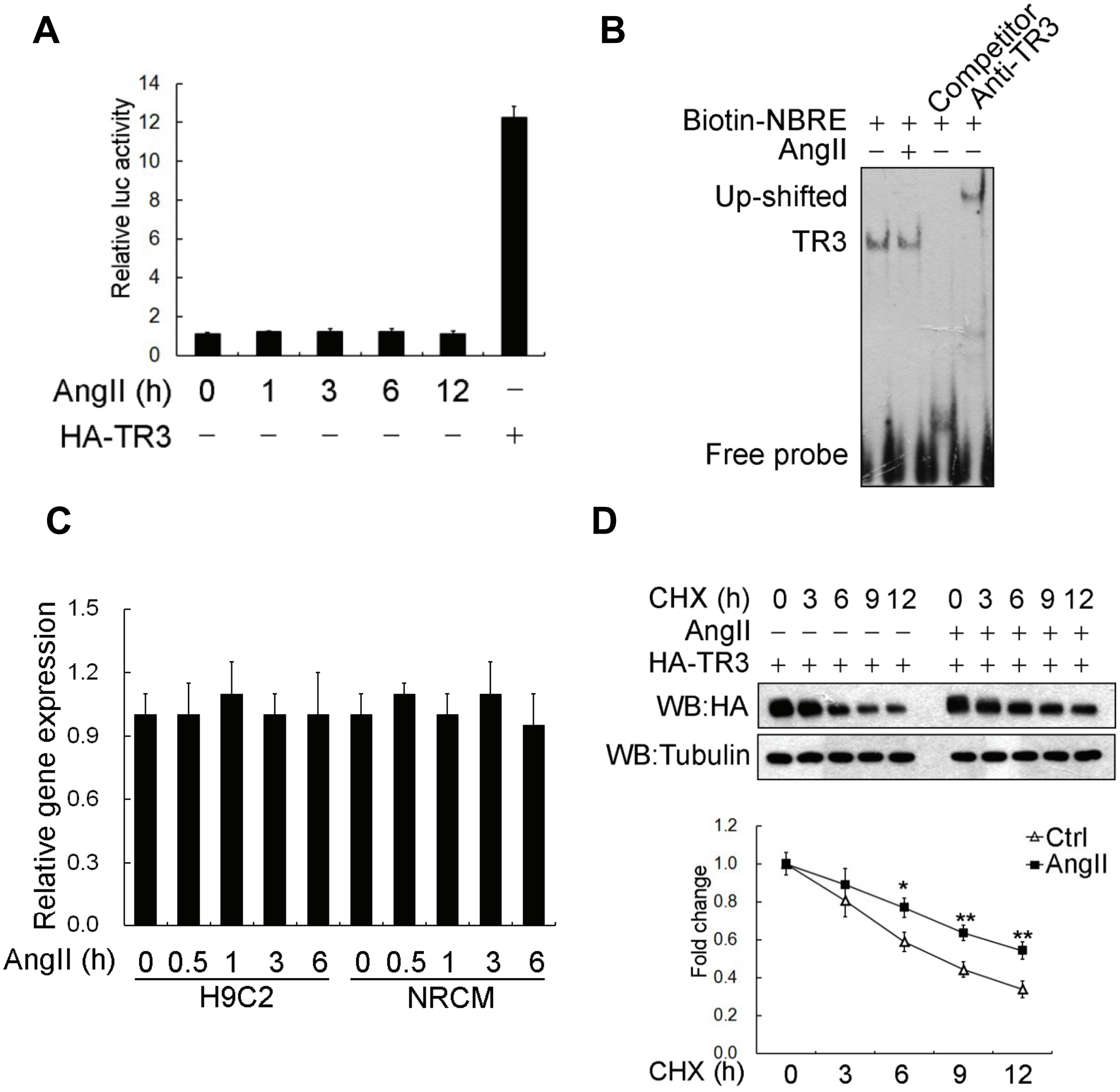

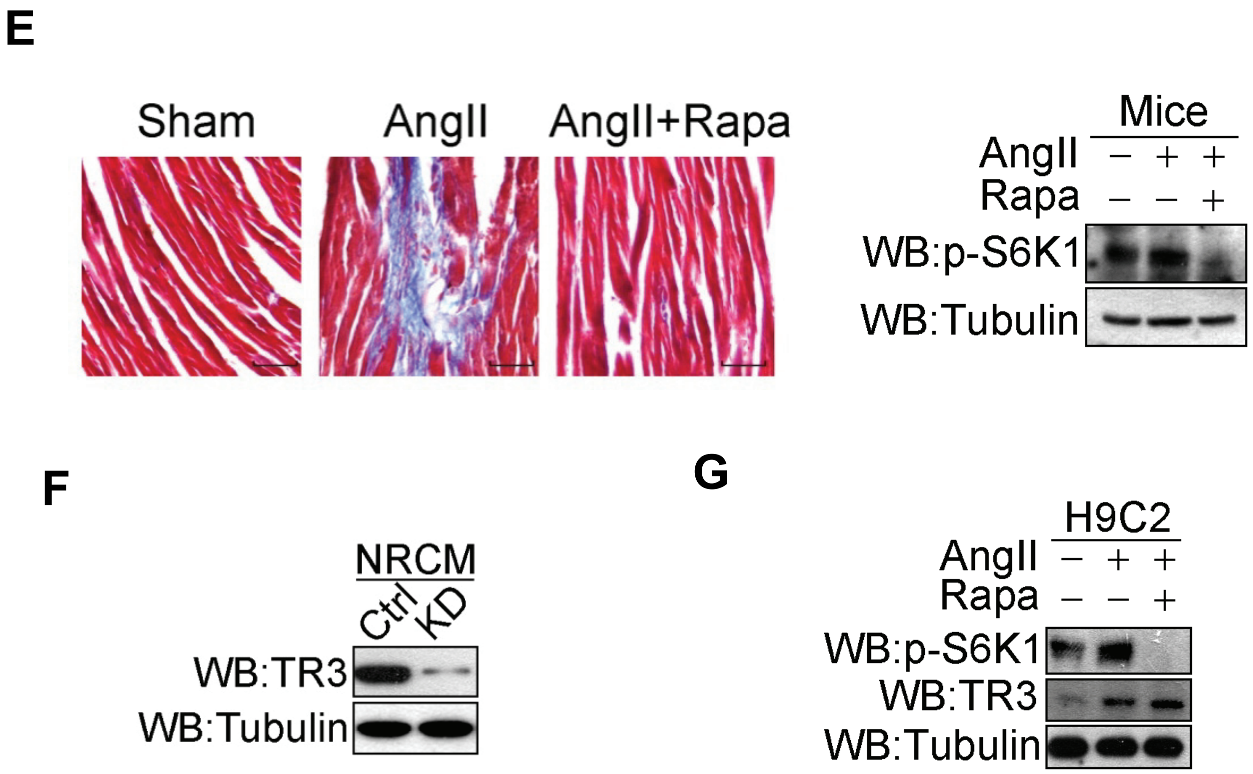
E
Sham
AngII
AngII+Rapa
Mice
AngII − + +
Rapa − − +
WB:p-S6K1
WB:Tubulin
F
NRCM
Ctrl
KD
WB:TR3
WB:Tubulin
G
H9C2
AngII − + +
Rapa − − +
WB:p-S6K1
WB:TR3
WB:Tubulin

Supplementary Fig. S4

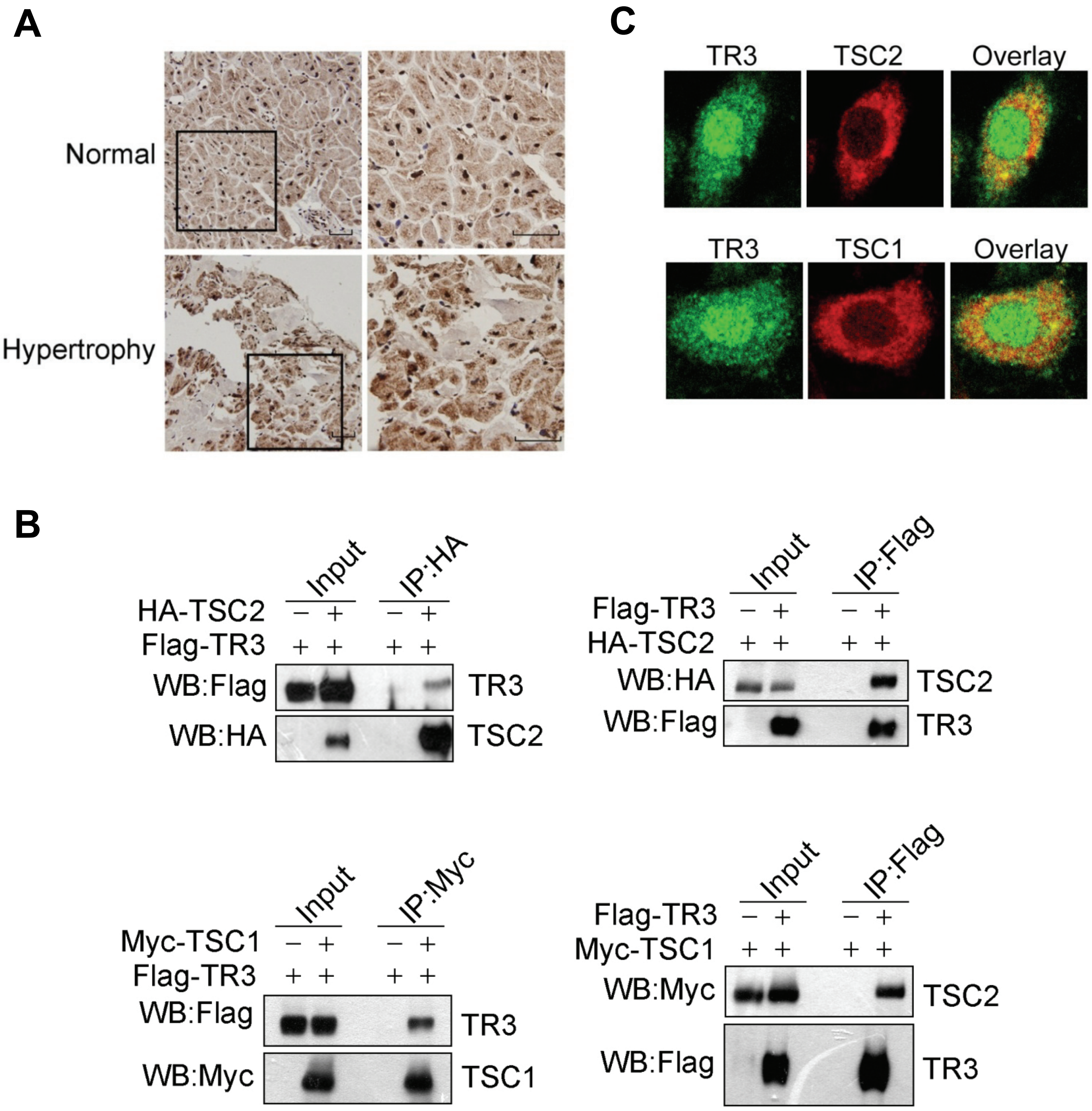

D

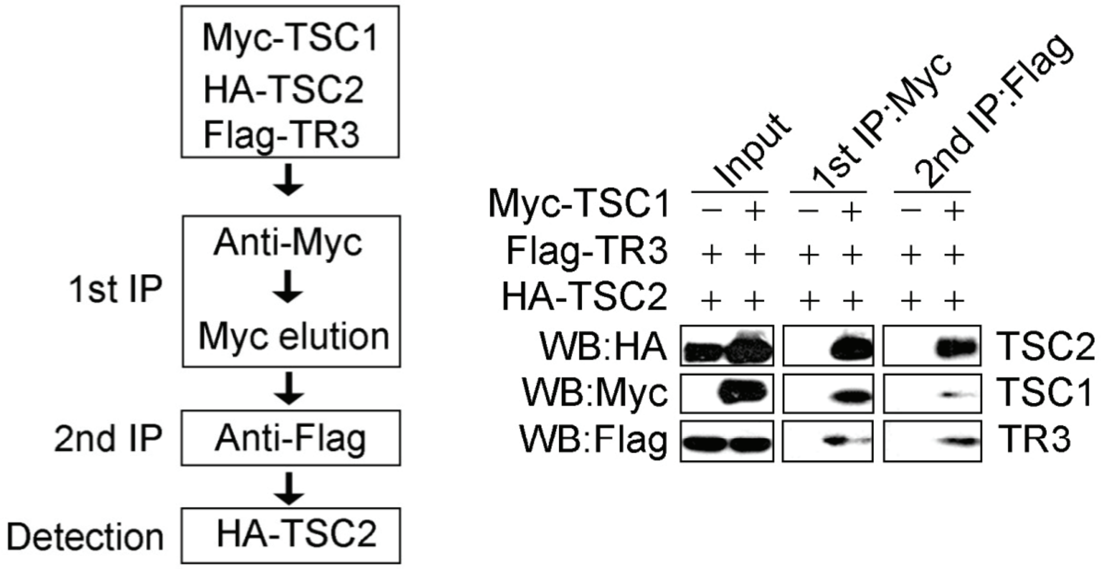
Myc-TSC1
HA-TSC2
Flag-TR3
1st IP
Anti-Myc
Myc elution
2nd IP
Anti-Flag
Detection
HA-TSC2
Input
1st IP:Myc
2nd IP:Flag
Myc-TSC1 − + − + − +
Flag-TR3 + + + + + +
HA-TSC2 + + + + + +
WB:HA
WB:Myc
WB:Flag
TSC2
TSC1
TR3

E

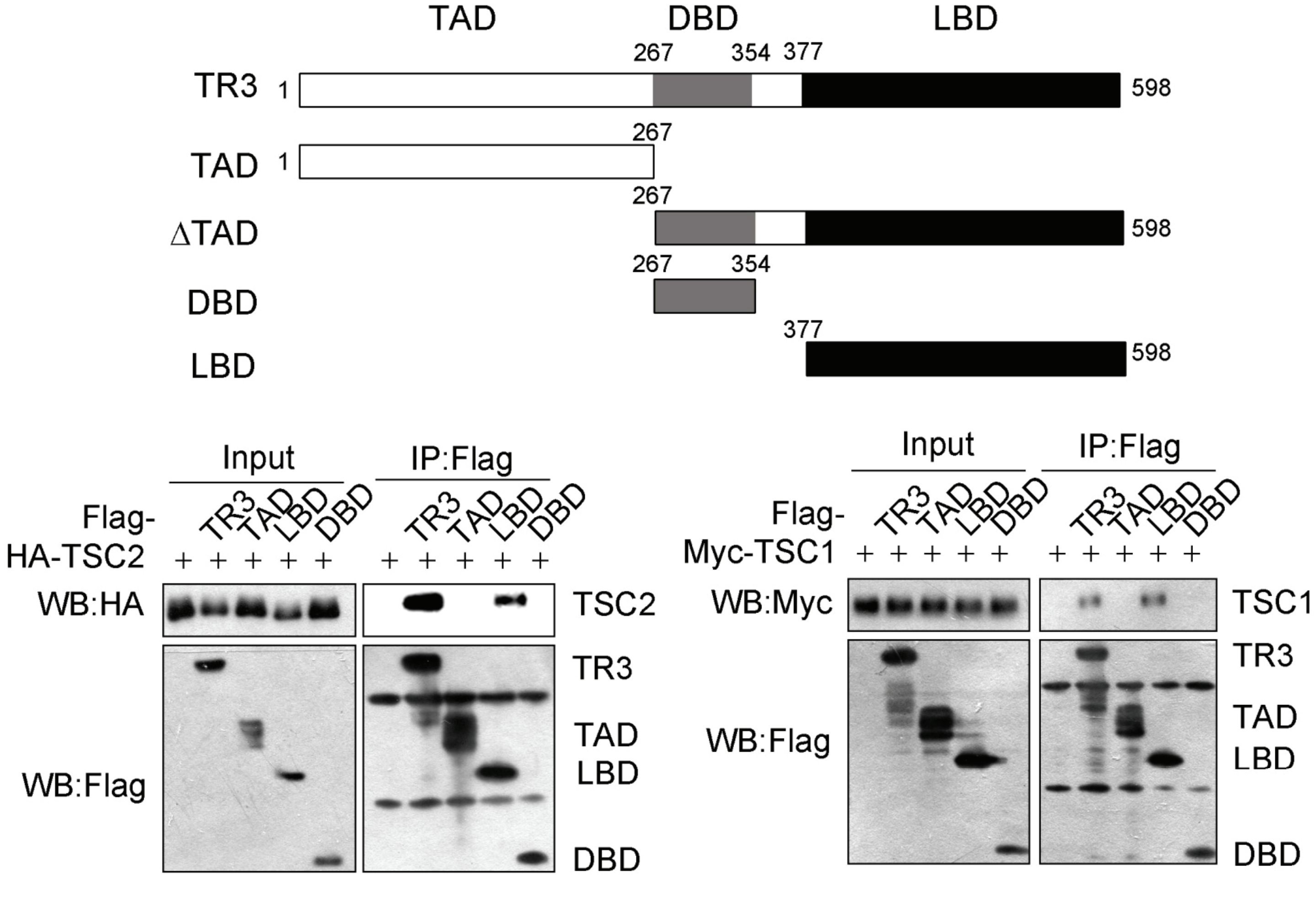
TAD
DBD
LBD
TR3
1
267
354
377
598
TAD
ΔTAD
DBD
LBD
Input
IP:Flag
Flag-
TR3 TAD LBD DBD
HA-TSC2
WB:HA
WB:Flag
TSC2
TR3
TAD
LBD
DBD
Myc-TSC1
WB:Myc
TSC1

Supplementary Fig. S5

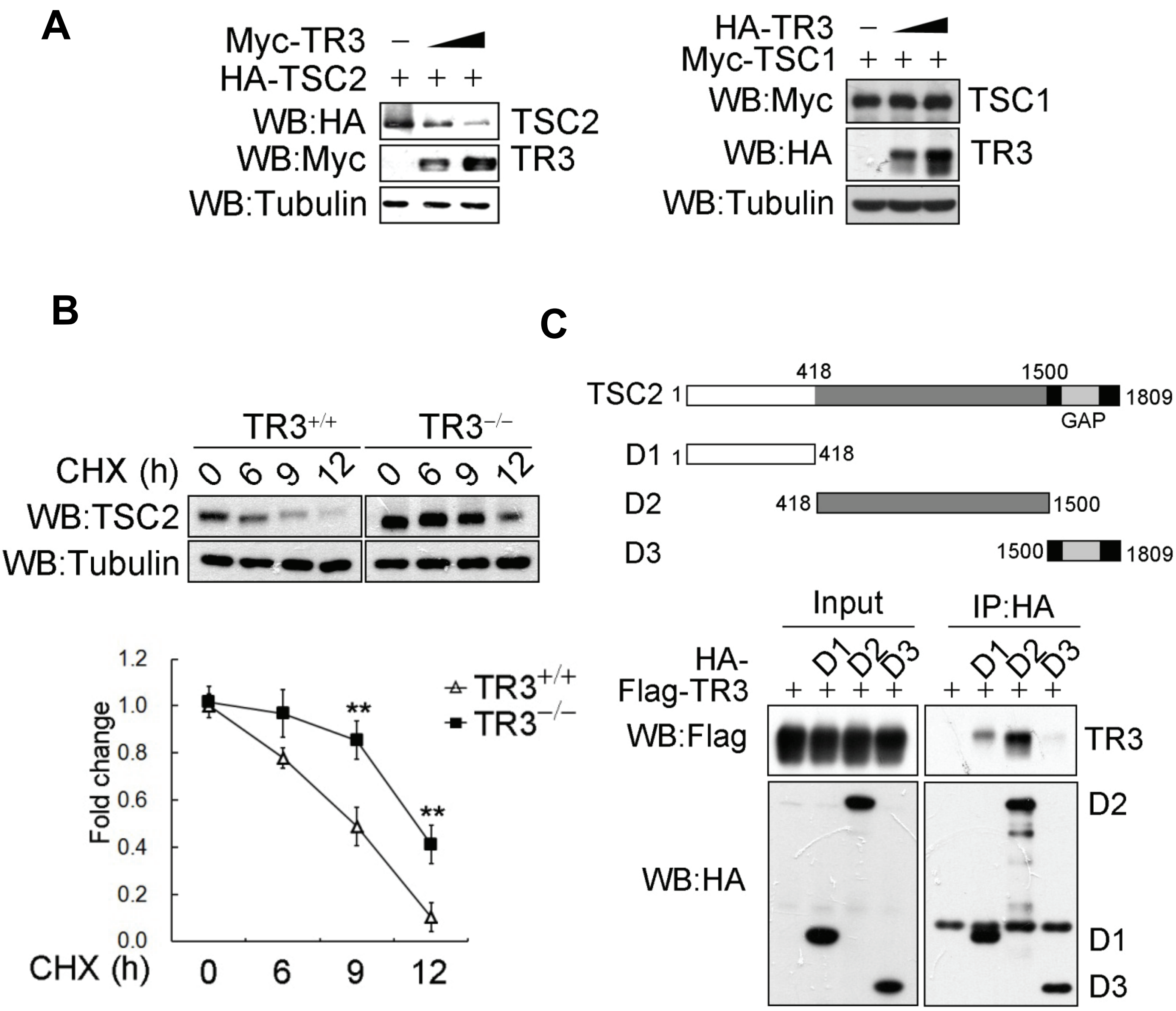

Supplementary Fig. S6

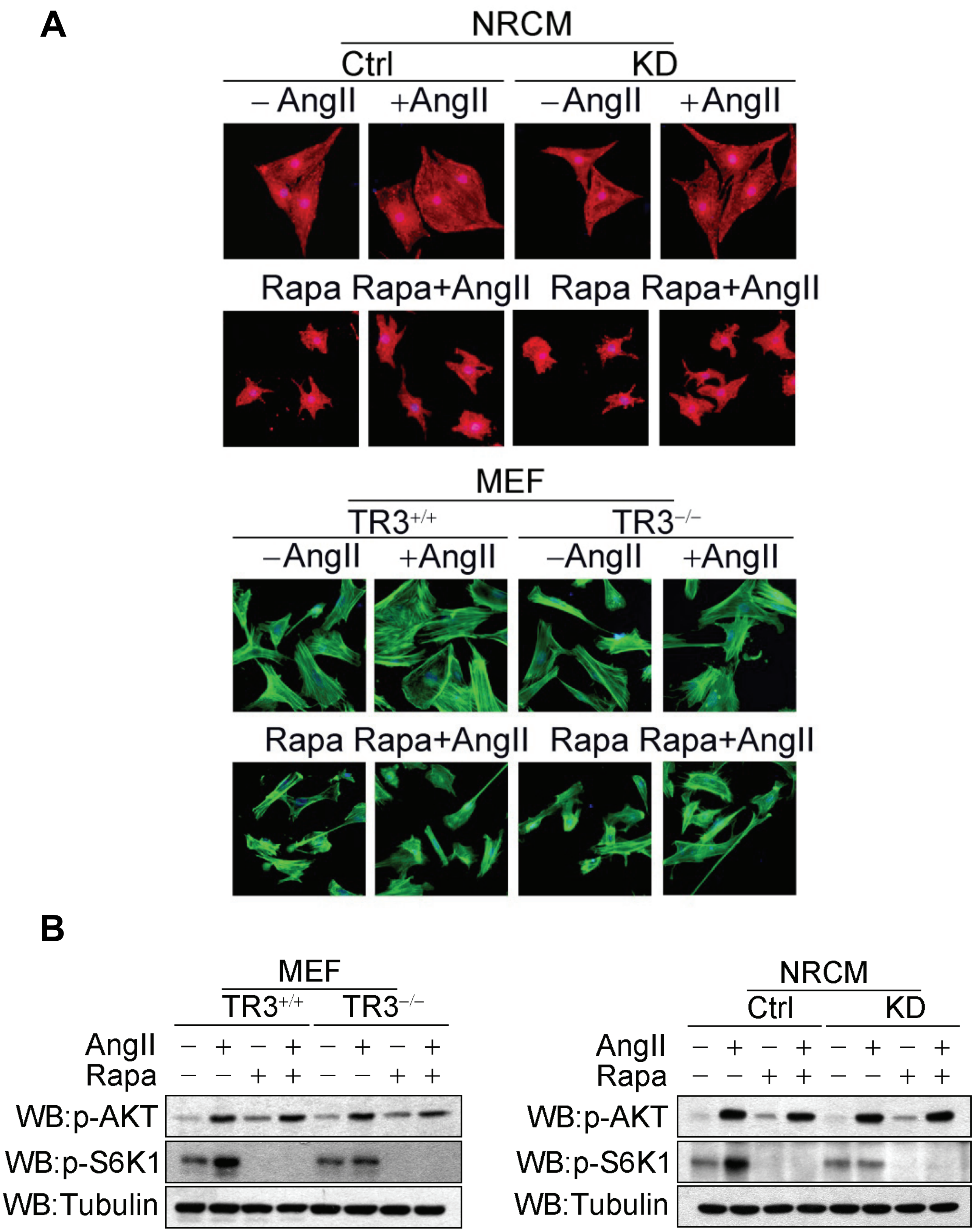

Supplement: Supplementary file 2 [file emmm0005-0137-SD2.pdf]
